# Molecular Mechanisms of Obesity-Associated Oxidative Stress and Therapeutic Strategies

**DOI:** 10.3390/antiox15091193

**Published:** 2026-09-18

**Authors:** Rongyu Wang, Yingyu Luo, Liying Liu, Jingwen Zhang, Rong Li, Nannan Zhang

**Affiliations:** 1 Department of Traditional Chinese Medicine, West China Second University Hospital, Sichuan University, Chengdu 610041, China; rongyu-wang@scu.edu.cn (R.W.);; 2 Key Laboratory of Birth Defects and Related Diseases of Women and Children, Sichuan University, Ministry of Education, Chengdu 610041, China; 3 National Center for Birth Defect Monitoring, West China Second University Hospital, Sichuan University, Chengdu 610041, China; 4 Acupuncture and Tuina School, Chengdu University of Traditional Chinese Medicine, Chengdu 610075, China; 5 Antibiotics Research and Re-Evaluation Key Laboratory of Sichuan Province, Sichuan Industrial Institute of Antibiotics, School of Pharmacy, Chengdu University, Chengdu 610106, China

**Keywords:** obesity, oxidative stress, adipose tissue, mitochondrial, endoplasmic reticulum

## Abstract

Obesity is a chronic metabolic disorder characterized by excessive adipose tissue accumulation and associated with systemic oxidative stress, mitochondrial dysfunction, and chronic low-grade inflammation. Adipose tissue dysfunction promotes the overproduction of reactive oxygen species through multiple mechanisms, including NADPH oxidase activation, mitochondrial electron transport chain leakage, and endoplasmic reticulum stress via mitochondria-associated ER membranes. The resulting oxidative stress damages lipids, proteins, and DNA, while activating redox-sensitive signaling pathways that perpetuate inflammation and insulin resistance. Moreover, obesity impairs endogenous antioxidant defense systems, including reduced activity of superoxide dismutase, glutathione peroxidase, and catalase, as well as magnesium deficiency that compromises glutathione synthesis. This review synthesizes current knowledge of molecular mechanisms linking obesity to oxidative stress and discusses key biomarker categories, including lipid peroxidation products, protein oxidation markers, DNA damage markers, adipokine ratios, and microRNAs. We also evaluate therapeutic strategies targeting obesity-associated oxidative stress, with emphasis on lifestyle interventions, pharmacological agents, and dietary compounds including resveratrol, curcumin, lycopene, and astaxanthin. Understanding the relationship between obesity and oxidative stress is essential for developing mechanism-directed therapeutic interventions.

## 1. Introduction

The global prevalence of obesity has risen dramatically over the past four decades, reaching pandemic proportions and imposing an immense burden on public health systems worldwide. According to the World Health Organization (2022, updated 2025) [1], 2.5 billion adults aged ≥18 years were overweight, including 890 million with obesity, corresponding to 43% and 16% of the adult population, respectively [1]. The Lancet estimates that over 2.11 billion adults were overweight or obese in 2021, with the prevalence of obesity more than doubling since 1990 [2]. Obesity is a major risk factor for cardiovascular diseases, type 2 diabetes mellitus (T2DM), non-alcoholic fatty liver disease, and several cancers, posing a substantial burden on global public health [3,4].

Adipose tissue (AT), historically viewed as a passive energy storage depot, is now recognized as a dynamic endocrine organ that orchestrates systemic metabolic homeostasis through the secretion of diverse adipokines [5]. In obesity, excessive AT expansion triggers chronic low-grade inflammation, characterized by elevated levels of pro-inflammatory mediators such as tumor necrosis factor-α (TNF-α), interleukin-6 (IL-6), and interleukin-1β (IL-1β), which are primarily produced by hypertrophic adipocytes and infiltrating immune cells [6,7]. Concurrently, oxidative stress, defined as a disruption of cellular redox balance resulting from excessive reactive oxygen species (ROS) production overwhelming endogenous antioxidant defenses, has emerged as a central pathogenic mechanism linking obesity to its downstream metabolic complications [8,9,10]. Mitochondrial dysfunction, characterized by impaired oxidative phosphorylation, increased electron leakage, and disrupted dynamics, serves as a major source of ROS in obesity, broadly affecting AT, skeletal muscle, liver, and the central nervous system [11,12,13,14,15,16,17,18,19,20]. Additionally, magnesium deficiency, commonly observed in obesity, may compromise endogenous antioxidant capacity [21,22,23,24], and recent evidence has extended the scope of obesity-associated stress to the maternal-placental-fetal unit, where placental oxidative stress contributes to fetal programming of metabolic disease [25,26,27]. Furthermore, the physical and functional crosstalk between mitochondria and the endoplasmic reticulum (ER) via mitochondria-associated ER membranes (MAMs) adds another layer of complexity to obesity-induced cellular stress [28]. In this context, obesity-associated oxidative stress can be conceptualized as an integrated network in which nutrient overload, mitochondrial dysfunction, and impaired antioxidant adaptation function as interdependent drivers that sustain and exacerbate oxidative damage across multiple tissues.

While previous reviews have comprehensively described individual pathways, including mitochondrial dysfunction, inflammation, ER stress, and antioxidant defense failure, most have treated these as parallel mechanisms without systematically integrating their synergistic and bidirectional interactions. Furthermore, the majority have focused on AT or the liver in isolation, with limited attention to interorgan amplification loops or to emerging mechanisms. Several reviews published in the past five years on obesity and oxidative stress illustrate these limitations, yet they differ in scope and emphasis. A review exploring how lipid storage drives AT expansion and how hypertrophy promotes oxidative stress, inflammation, and insulin resistance (IR), while also evaluating lipids, oxidative stress markers, and inflammatory mediators as biomarkers of metabolic dysfunction and assessing the therapeutic potential of antioxidants, provides a thorough analysis of AT but does not examine the role of MAMs in ER-mitochondrial crosstalk [29]. An examination of the nexus between obesity and cognitive dysfunction in diabetes centers on neuroinflammation and IR but does not consider the propagation of peripheral oxidative stress from metabolic organs to the central nervous system [30]. A review elucidating how ROS exacerbate mitochondrial dysfunction, protein damage, and lipid peroxidation, and impair antioxidant function in the pathogenesis of metabolic syndrome, which comprises IR, hypertension, and hyperlipidemia, treats mitochondrial dysfunction and inflammation as largely tissue-autonomous processes, with limited integration of inter-organ communication [31]. A review focused on obesity-induced skeletal muscle IR confines its discussion of oxidative stress to skeletal muscle, without extending to upstream MAM signaling or crosstalk with liver and AT [32]. A review updating the pathophysiological mechanisms of obesity in relation to oxidative stress and emphasizing the impact of endogenous and exogenous antioxidants on the evolutionary course of pediatric obesity mentions placental oxidative stress only briefly, without mechanistic integration into core pathways of mitochondrial dysfunction and ER stress [33]. A systematic review of genetic variants of antioxidant enzymes, ROS generators, and transcription factors in relation to oxidative stress levels and the risk of obesity, IR, and metabolic syndrome does not address dynamic nuclear factor erythroid 2-related factor 2 (NRF2) regulation or the emerging role of ferroptosis in obesity-related oxidative stress [34]. Finally, a review systematically delineating how obesity triggers oxidative stress through metabolic, endocrine, inflammatory, and gut microbiota pathways to impair the endometrium does not extend to the multi-organ integrative framework proposed here, nor does it address the upstream regulatory role of MAMs [35].

To address these gaps, the present review provides a multi-level, inter-organ integrative framework that conceptualizes obesity-associated oxidative stress as an interdependent network of drivers in which nutrient excess and MAM-mediated ER-mitochondrial calcium crosstalk function as upstream triggers, ROS production and inflammation serve as core amplifiers, and antioxidant system failure with ferroptosis susceptibility represents downstream vulnerability nodes that propagate damage across tissues. We systematically examine the role of MAMs-mediated ER-mitochondrial calcium crosstalk as an upstream hub that integrates cellular stress responses, incorporate NRF2-regulated ferroptosis defense mechanisms into the framework of antioxidant system failure, and construct a cross-organ feed-forward amplification model tracing the propagation of oxidative damage from AT to the liver, skeletal muscle, central nervous system, and the maternal-placental-fetal unit. In addition, we critically discuss unresolved controversies, such as the dichotomous roles of NOX4 in adipocytes, the tissue- and context-dependent effects of NRF2 activation, and whether antioxidant system dysfunction is a cause or a consequence of obesity. We also explicitly distinguish between preclinical mechanistic findings and human observational data, thereby identifying translational limitations that have been underappreciated in prior syntheses. Through this integrative framework, we aim to provide a more systematic conceptual model for understanding the pathophysiology of obesity-associated oxidative stress and to inform mechanism-directed therapeutic strategies.

## 2. Molecular Mechanisms of Obesity-Associated Oxidative Stress

### 2.1. Mitochondrial Dysfunction-Mediated Oxidative Stress

**Figure 1:**
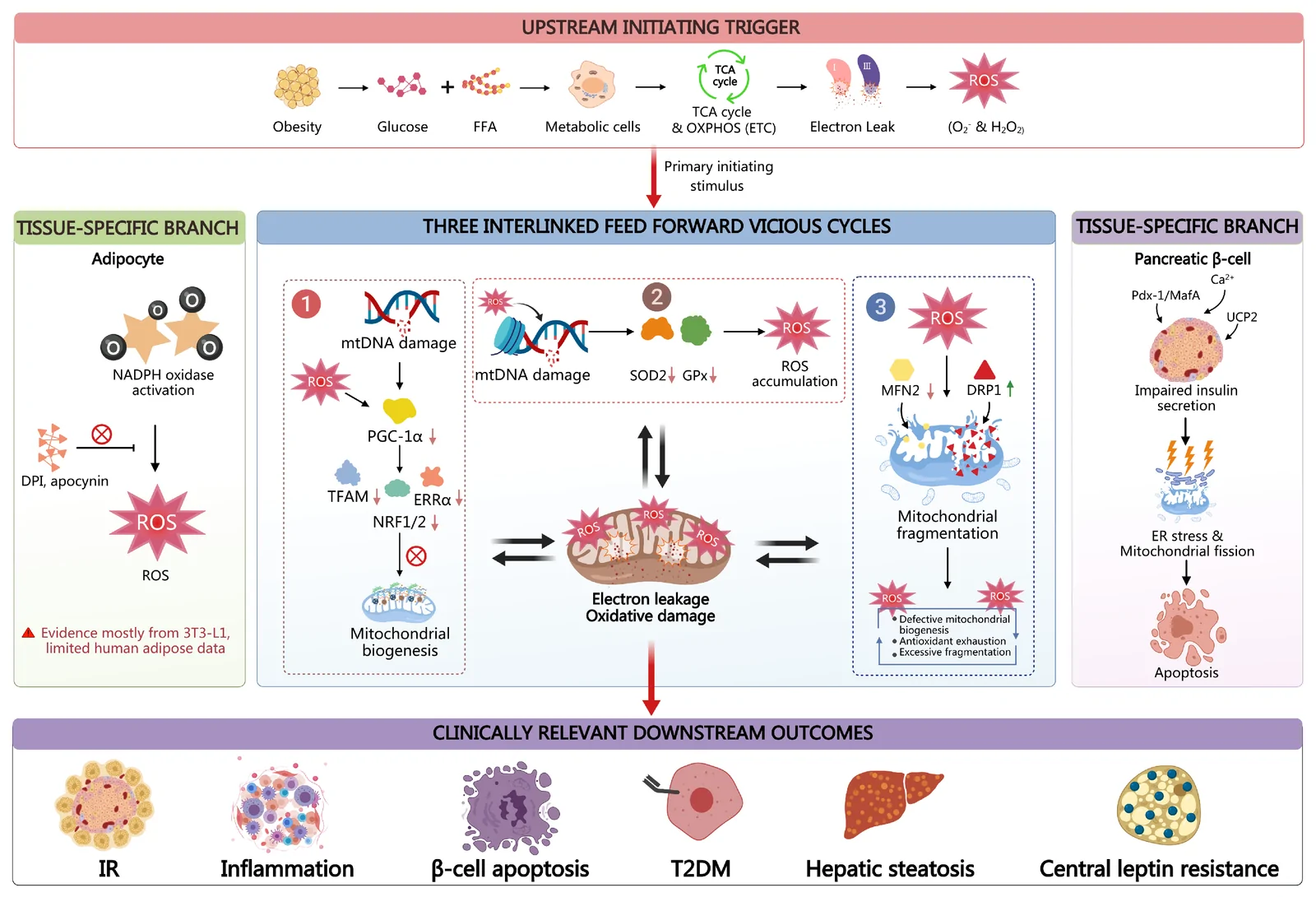
Mitochondrial dysfunction-mediated oxidative stress in obesity.

Mitochondria serve as the primary site of intracellular ATP production and the major cellular source of ROS, placing these organelles at the core intersection of systemic energy metabolism and redox homeostasis [11,12]. Disrupted mitochondrial redox balance constitutes the foundational driver of obesity-associated oxidative stress, as illustrated in Figure 1. Mitochondrial ATP synthesis relies on coordinated electron transfer across four inner-membrane electron transport chain (ETC) complexes during oxidative phosphorylation [36]. Under normal conditions, less than 5% of electrons leak from the ETC to generate superoxide anions (O_2_^−^), which are rapidly neutralized by endogenous antioxidant systems [37]. However, chronic nutrient overload in obesity delivers sustained excess glucose and free fatty acids (FFAs) to mitochondrial compartments, overloading both the tricarboxylic acid (TCA) cycle and ETC flux. This metabolic pressure elevates electron leakage rates and substantially boosts the generation of O_2_^−^, hydrogen peroxide (H_2_O_2_), and other cytotoxic ROS throughout metabolically active tissues [38,39].

Chronic overexposure to glucose and FFA in obesity overloads the TCA cycle and ETC, triggering excessive electron leakage at complexes I/III and ROS overproduction. Accumulated ROS initiates three interlinked feedforward vicious cycles that mutually amplify mitochondrial impairment: (1) ROS-mediated mtDNA damage suppresses the PGC-1α/NRF1/2/ERRα/TFAM axis to block mitochondrial biogenesis, further aggravating ETC dysfunction and ROS generation; (2) oxidative lesions to mtDNA reduce expression and activity of antioxidant enzymes SOD2 and GPx, ablating mitochondrial ROS clearance and sustaining redox imbalance; (3) ROS downregulates fusion mediator MFN2 while promoting DRP1 recruitment to mitochondria via Fis1/Mff/MiD49/MiD50, driving pathological mitochondrial fragmentation that inhibits complex III activity and elevates ROS release in turn. These synergistic self-amplifying redox loops converge to reduce Δψ_m_, cristae density, and mtDNA copy number, ultimately propagating IR and systemic inflammation. Tissue-specific divergent pathogenic branches are delineated: adipocyte ROS production predominantly depends on NOX activation, which is suppressed by DPI or apocynin, whereas pancreatic β-cells exhibit impaired insulin secretion via UCP2 induction, Pdx-1/MafA suppression, and disrupted calcium homeostasis, with mitochondrial fragmentation cooperating with ER stress to promote β-cell apoptosis. Most mechanistic observations are derived from preclinical cell and rodent models, while sufficient human AT validation remains lacking. Collectively, the above cascades accelerate β-cell loss and advance obesity-related metabolic disorders. (FFA, free fatty acids; TCA, tricarboxylic acid; ETC, electron transport chain; ROS, reactive oxygen species; mtDNA, mitochondrial DNA; PGC-1α, peroxisome proliferator-activated receptor gamma coactivator 1-α; NRF1/2, nuclear respiratory factors 1/2; ERRα, estrogen-related receptor α; TFAM, mitochondrial transcription factor A; SOD2, superoxide dismutase 2; GPx, glutathione peroxidase; MFN2, mitofusin 2; DRP1, dynamin-related protein 1; Δψ_m_, mitochondrial membrane potential; IR, insulin resistance; NOX, NADPH oxidase; UCP2, uncoupling protein 2; ER, endoplasmic reticulum).

#### 2.1.1. Structural and Functional Impairment of Mitochondria

Chronic exposure to surplus FFAs secreted from hypertrophic visceral adipocytes triggers lipotoxic injury to mitochondrial architecture, disrupting membrane integrity, diminishing cristae density, and inducing organelle swelling across adipose, hepatic, and muscle tissues [40,41,42,43]. In vitro cellular assays using differentiated 3T3-L1 adipocytes confirm that FFA exposure alone reproduces mitochondrial cristae fragmentation, swelling, and mtDNA lesions accompanied by robust ROS accumulation, with ETC complex catalytic activity negatively correlated with intracellular ROS abundance [44,45]. However, the translational relevance of these findings is constrained by important methodological considerations. Differentiated 3T3-L1 adipocytes are immortalized cells derived from a single clonal line that may not fully recapitulate the metabolic complexity of primary adipocytes [46]. Furthermore, the supraphysiological FFA concentrations often range from 1500 to 4000 μmol/L, substantially exceeding those encountered in vivo, where circulating levels typically remain below 800 μmol/L even under obese or IR conditions [47]. This discrepancy raises questions regarding the physiological relevance of the observed effects. In high-fat diet (HFD)-induced obese mice, Δψ_m_ is significantly reduced in visceral adipose tissue (VAT) and liver, while ROS levels are twice as high as those in normal controls [48,49,50,51]. Human observational cohorts extend these preclinical observations that compromised mitochondrial oxidative capacity in the skeletal muscle of individuals with obesity or T2DM is associated with reduced activity of ETC complexes I, III, and IV and lower substrate oxidation rates compared with lean controls [52,53,54,55]. These findings are important because they confirm that the mitochondrial defects observed in cell culture and rodents are recapitulated in human disease. However, the interpretative power of human studies is constrained by their observational design. Cross-sectional associations cannot distinguish whether mitochondrial dysfunction precedes obesity and drives metabolic deterioration or whether it emerges as a secondary consequence of prolonged metabolic stress. A depolarized Δψ_m_ further disrupts sequential electron transfer along the ETC, accelerating electron leakage at complexes I and III and sustaining persistent ROS overproduction [13,56,57]. Tissue-specific metabolic consequences of mitochondrial dysfunction vary across organ systems: in skeletal muscle, excessive ROS and attenuated ATP synthesis directly correlate with IR [14,18]; in the liver, mitochondria-derived ROS drive lipid peroxidation and accelerate pathological progression from simple hepatic steatosis to irreversible fibrosis [19]. White adipose tissue (WAT) from obese patients also presents depleted mitochondrial abundance, reduced mtDNA copy numbers, and global downregulation of oxidative phosphorylation transcripts, while the severity of hepatic mitochondrial impairment varies dynamically with obesity duration and disease severity [58,59,60]. The patterns of mitochondrial impairment thus differ across organs, including tissue-specific FFA accumulation capacity, baseline mitochondrial substrate preference, and intrinsic differences in mitochondrial proteome composition. The mechanistic basis of this heterogeneity remains poorly understood, as comparative studies across tissues from the same individuals are rare. Suppressed mitochondrial biogenesis represents a central upstream contributor to widespread mitochondrial structural and functional defects in obesity. Peroxisome proliferator-activated receptor gamma coactivator 1-α (PGC-1α) acts as the master transcriptional regulator of mitochondrial biogenesis, sequentially activating nuclear respiratory factors (NRF1/2) and estrogen-related receptor α (ERRα), which in turn induce mitochondrial transcription factor A (TFAM) to support mtDNA replication and transcription of mitochondrially encoded ETC subunits [61,62]. In both human obesity and animal models, PGC-1α expression is markedly reduced in AT, which correlates with impaired mitochondrial biogenesis, reduced oxidative phosphorylation capacity, and subsequent IR [56,63,64]. Nevertheless, the correlational nature of these observations in human studies leaves unanswered the question of whether PGC-1α downregulation is a primary defect predisposing to obesity or an adaptive response to chronic nutrient excess. In summary, the current evidence establishes that FFA-induced mitochondrial injury occurs across experimental systems. However, the causal primacy of mitochondrial dysfunction in human obesity remains unproven due to the correlational nature of human data, the predominant use of male rodents, and the limited availability of prospective longitudinal studies. Resolving these questions will require well-designed human studies that can distinguish causation from correlation and integrate multiple tissue types and longitudinal design.

#### 2.1.2. Imbalanced Mitochondrial Dynamics and the Vicious Cycle

Mitochondrial quality control depends on continuous fusion and fission cycles, which facilitate adaptive remodeling in response to fluctuating metabolic demand, enable inter-mitochondrial metabolite exchange, and segregate irreversibly damaged organelles for autophagic clearance [65]. Mitochondrial fusion is coordinated by outer-membrane mitofusin 1 (MFN1) and mitofusin 2 (MFN2), alongside inner-membrane optic atrophy 1 (OPA1) [66], while fission initiation requires cytosolic dynamin-related protein 1 (DRP1), recruited to the mitochondrial outer membrane via auxiliary receptors including Fis1, Mff, MiD49, and MiD50 [67]. Obesity profoundly shifts this homeostatic balance toward excessive mitochondrial fragmentation, with consistent phenotypes observed across preclinical and human samples. In HFD-fed mice, skeletal muscle exhibits smaller, more fragmented mitochondria with increased DRP1-mediated fission activity [68]. Human observational data complement these findings, showing significantly reduced MFN2 protein abundance in skeletal muscle biopsies from obese and diabetic individuals, and its expression level correlates positively with insulin sensitivity and inversely with BMI [69,70]. However, the apparent consistency of these findings masks several interpretative challenges that are not yet fully resolved. First, the direction of causality remains unresolved. While animal studies suggest that DRP1 upregulation precedes metabolic dysfunction, human cross-sectional data cannot distinguish whether altered mitochondrial dynamics drive IR or represent a compensatory response to metabolic stress. Second, the tissue specificity of these changes is poorly characterized. Most human studies have focused on skeletal muscle due to biopsy accessibility, yet the relevance of muscle mitochondrial dynamics to systemic IR may differ from that of AT or the liver. Whether similar MFN2 reductions and DRP1 elevations occur in human AT or hepatocytes remains to be systematically characterized. Third, contradictory findings have emerged regarding the functional consequences of altered mitochondrial dynamics. DRP1 inhibition in cell-based models reduces ROS production and improves insulin sensitivity, suggesting that excessive fission is pathogenic. However, MFN2 haploinsufficiency also impairs mitochondrial function [71], indicating that insufficient fusion can be equally detrimental. These opposing observations suggest that the relationship between mitochondrial dynamics and metabolic health is not linear: moderate fission may be required for quality control, whereas excessive fission and insufficient fusion are pathogenic. The threshold at which fission transitions from beneficial to detrimental has not been defined in any tissue. Fourth, the specificity of the experimental tools used to manipulate mitochondrial dynamics warrants scrutiny. Pharmacological DRP1 inhibitors such as Mdivi-1 have been widely employed to demonstrate the pathogenic role of fission, yet Mdivi-1 also affects mitochondrial complex I activity and may have off-target effects that confound interpretation. Genetic approaches, while more specific, have yielded inconsistent results depending on the timing and cell type of DRP1 deletion, suggesting that the role of fission may be context-dependent. Functionally, unregulated mitochondrial fission reduces complex III catalytic activity by over 30%, suppresses the expression and activity of cytosolic and mitochondrial superoxide dismutases (SOD1, SOD2), and exacerbates mtDNA oxidative lesions [72,73]. This excessive fission promotes ROS production, impairs oxidative phosphorylation, and sensitizes cells to apoptosis. In turn, ROS further damages ETC components and mtDNA, establishing a self-reinforcing cycle that amplifies mitochondrial dysfunction and activates inflammatory pathways. Clinical observations further contextualize this cycle. Patients with metabolic syndrome exhibit far more severe mitochondrial dysfunction and systemic oxidative stress than individuals with simple obesity, and sustained weight loss partially restores mitochondrial structure and redox homeostasis [74]. However, these correlative clinical outcomes cannot confirm direct causal mediation by mitochondrial fragmentation. Obesity disrupts mitochondrial dynamics by downregulating Mfn1/2 and upregulating Drp1, thereby amplifying ROS release and creating a vicious cycle of mitochondrial dysfunction that drives inflammation and IR [14,15].

#### 2.1.3. Failure of the Antioxidant Defense System

Mitochondria possess a specialized, compartmentalized antioxidant network comprising SOD2, glutathione peroxidase (GPx), and catalase, which constrains basal ROS concentrations to levels compatible with physiological redox signaling [75]. In the HFD-induced obesity model, chronic ROS exposure damages mtDNA, which encodes ETC subunits and key antioxidant enzymes (such as SOD2), thereby reducing their expression and activity and stimulating further ROS accumulation [56,76,77]. Damaged mtDNA leads to decreased SOD2 and GPx expression, with corresponding reductions in enzyme activity, thereby limiting the ability to adequately scavenge excessive ROS, resulting in ROS accumulation, particularly within the mitochondrial matrix [56,78]. Multiple peripheral tissues, including cardiac muscle and circulating blood cells, exhibit suppressed SOD2 activity in HFD-fed mice, accompanied by elevated systemic oxidative damage biomarkers, such as malondialdehyde (MDA), and heightened mitochondrial ROS accumulation [79,80,81]. This failure creates a second vicious cycle, in which excessive ROS leads to mtDNA damage, which in turn reduces antioxidant enzyme activity and permits further ROS accumulation. Additionally, obesity-induced IR inhibits AMPK activation, leading to abnormal transcriptional regulation of mitochondrial antioxidant enzymes and further aggravating oxidative stress [82]. However, translating this model to human obesity raises several unresolved issues. First, quantifying mtDNA damage in human tissues is methodologically challenging. The most commonly used approach, long-range PCR, is susceptible to artifacts arising from DNA fragmentation during sample processing, and the results can vary substantially depending on DNA extraction methods and primer design [83]. Alternative approaches, such as next-generation sequencing, offer greater resolution, but their clinical application has not yet been systematically evaluated. Consequently, the reported association between mtDNA damage and obesity in humans should be interpreted with caution, as the methodological heterogeneity across studies may complicate cross-study comparison. Second, the causal direction of the relationship between mtDNA damage and antioxidant enzyme decline has not been established in humans [84]. Preclinical studies using cell culture and rodent models have demonstrated that ROS exposure causes mtDNA damage and reduces SOD2 expression, but whether this sequence of events occurs in human obesity has not been tested prospectively. No longitudinal study has tracked mtDNA integrity and SOD2 activity before and after weight gain in the same individuals. Third, the contribution of mtDNA damage to systemic metabolic dysfunction remains uncertain. While correlations between mtDNA copy number (a proxy for mitochondrial content) and IR have been reported in multiple cohorts [85,86], these associations do not establish causality. In addition, mtDNA copy number is influenced by multiple factors, including age and sex, and is correlated with hematological parameters, specifically a positive association with platelet count and a negative association with white blood cell count [87,88]. Although these confounders are well documented, cross-sectional studies investigating the association between mtDNA copy number and metabolic outcomes often fail to adequately adjust for them, potentially introducing residual confounding and contributing to the inconsistent findings reported across studies. Fourth, the relative importance of the mtDNA damage cycle versus other ROS-generating mechanisms, such as NOX activation and ETC leakage, has not been quantified in any tissue. It is possible that the mtDNA damage cycle operates as an amplifying mechanism rather than a primary driver, exacerbating oxidative stress initiated by other pathways. This distinction has therapeutic implications: targeting the cycle may be beneficial as an adjunctive strategy but insufficient as a standalone intervention.

A prominent research gap exists in translational human studies investigating the mechanistic link between mtDNA damage and mitochondrial antioxidant failure. While comprehensive mechanistic evidence linking mtDNA mutations to ROS production and antioxidant dysfunction derives predominantly from preclinical rodent models and cell culture systems, direct human tissue-specific evidence remains limited. In summary, the mtDNA damage-antioxidant failure cycle is conceptually compelling and supported by robust preclinical evidence, but human validation remains incomplete. Future studies should prioritize standardized mtDNA damage assays, prospective longitudinal designs, and multi-tissue analyses to establish the clinical relevance of this cycle in human obesity.

#### 2.1.4. An Alternative Pathway: NADPH Oxidase in Adipocyte ROS Production

In AT, ROS overproduction impairs adipocyte function and triggers lipotoxicity and inflammation [16,17]. The mitochondrial ETC is not the sole or primary source of FFA-induced ROS [89]. In differentiated 3T3-L1 adipocytes, linoleic acid-induced ROS production is completely blocked by NADPH oxidase (NOX) inhibitors (DPI, apocynin) but not by xanthine oxidase or mitochondrial complex inhibitors, indicating that FFAs in adipocytes promote oxidative stress primarily via NOX activation rather than through mitochondrial electron leakage [90]. However, several factors limit the generalizability of this conclusion. The observation that NOX inhibitors block FFA-induced ROS in 3T3-L1 adipocytes does not establish that NOX is the primary ROS source in all adipocyte populations or in intact AT. 3T3-L1 cells may exhibit NOX expression patterns distinct from primary adipocytes. Second, the inhibitors used in these studies, DPI and apocynin, are not entirely selective for NOX. DPI also inhibits mitochondrial complex I and other flavin-containing oxidoreductases, while apocynin has been shown to have antioxidant properties independent of NOX inhibition. These off-target effects complicate the interpretation of inhibitor-based experiments. Moreover, the NOX inhibitor experiments cited above were conducted in cell culture; whether similar results would be obtained in human AT explants or in vivo remains unknown. Although it remains unclear whether mitochondrial dysfunction is a cause or a consequence of IR and T2DM, improving mitochondrial function has been shown to ameliorate IR in multiple tissues, including skeletal muscle, liver, and AT [91,92,93]. In obese patients with IR, mitochondrial metabolic and biosynthetic pathways are markedly downregulated. These changes occur at both mRNA and protein levels, accompanied by reduced enzyme activities and mitochondrial density [54]. For instance, a monozygotic twin-pair study demonstrated that metabolically unhealthy obese individuals exhibit significant downregulation of oxidative phosphorylation, fatty acid β-oxidation, and branched-chain amino acid catabolism pathways in subcutaneous AT compared with their genetically identical lean co-twins. In contrast, metabolically healthy obese subjects maintain normal expression of these pathways [94]. This twin design, which controls for genetic background and early-life environmental factors, provides stronger evidence for acquired metabolic dysfunction than conventional case–control studies. Nevertheless, it remains unclear whether the observed downregulation represents a cause or a consequence of the unhealthy metabolic phenotype, as the cross-sectional nature of the study cannot establish temporal precedence. In pancreatic β-cells, IR and chronic hyperglycemia increase glucose and fatty acid metabolism, producing excess reducing equivalents (NADH and FADH_2_) that overdrive ETC activity. This promotes electron leakage—primarily at complexes I and III—generating superoxide and, via the Fenton reaction, highly reactive hydroxyl radicals [95]. β-cells are particularly vulnerable to this oxidative assault due to their intrinsically low expression of antioxidant enzymes (catalase and GPx) and high oxygen demand [96,97]. Accumulated ROS impair glucose-stimulated insulin secretion through activation of uncoupling protein 2 (UCP2), transcriptional downregulation of β-cell identity factors Pdx-1 and MafA, and disruption of intracellular calcium signaling, while simultaneously promoting β-cell apoptotic death via mitochondrial fission and concurrent ER stress [95]. Together, these mechanisms position mitochondrial dysfunction as a critical nexus in obesity-associated metabolic disease, linking obesity to oxidative stress and IR, with pancreatic β-cell dysfunction representing a key downstream consequence that accelerates progression to T2DM. In summary, NOX-mediated ROS production represents an important alternative pathway in adipocytes, but the evidence base is predominantly derived from cell culture studies, and the relative contribution of NOX versus mitochondrial ROS in human AT remains to be determined. Translating these preclinical insights to human obesity will require tissue-specific analyses in patient populations and careful consideration of context when designing therapeutic strategies.

### 2.2. Inflammation-Driven Oxidative Stress in Obesity

In addition to mitochondrial dysfunction, chronic low-grade inflammation is another pivotal driver of obesity-associated oxidative stress. The characteristic inflammatory state in obesity originates primarily from the expansion of AT. Chronic nutrient excess leads to insufficient angiogenesis and limited oxygen diffusion, thereby inducing relative hypoxia in hypertrophied adipocytes and hyperplastic AT [98]. This hypoxic environment inhibits the degradation of Hypoxia-Inducible Factor 1-α (HIF-1α), leading to its accumulation and the promotion of numerous pro-inflammatory gene expressions [99].

#### 2.2.1. Sources and Characteristics of Chronic Low-Grade Inflammation

AT from lean individuals contains a sparse immune cell compartment, with macrophages accounting for approximately 5% of total stromal cells and predominantly polarized toward an anti-inflammatory M2 phenotype defined by arginase-1 expression and secretion of anti-inflammatory cytokines [100]. Obesity dramatically alters this cellular landscape. The number of AT macrophages increases significantly and can account for up to 50% of total cells in some adipose depots [101]. This polarization transition is marked by elevated inducible nitric oxide synthase (iNOS) expression, enhanced secretion of TNF-α, IL-6, and IL-1β, and the formation of crown-like structures surrounding necrotic adipocytes [102]. While the qualitative features of this inflammatory transition are well-established, significant quantitative heterogeneity exists across studies. The reported proportion of AT macrophages in obesity ranges from 15% to 50% depending on the adipose depot examined, the method of quantification, and the obesity model [100,101,102]. This methodological heterogeneity complicates cross-study comparison and raises questions about whether a single “obesity-associated inflammatory phenotype” exists or whether distinct inflammatory subphenotypes are present in different patient populations. A more fundamental unresolved issue is the temporal relationship between macrophage infiltration and metabolic dysfunction. The conventional model posits that macrophage infiltration drives IR, but alternative interpretations are equally plausible. IR may precede and promote macrophage infiltration through adipocyte stress and lipolysis, or a third factor, such as genetic susceptibility or diet composition, may independently drive both phenomena. Obesity-associated inflammation differs from acute inflammation, which has overt clinical symptoms. It is characterized as “chronic low-grade” with persistently mild elevation of inflammatory levels [103]. These levels do not cause overt redness, swelling, heat, or pain but disrupt cellular homeostasis over the long term. Key pro-inflammatory factors involved include TNF-α, IL-6, IL-1β, and C-reactive protein (CRP) [104]. Clinical studies have shown that serum levels of these pro-inflammatory factors in obese individuals, particularly those with abdominal obesity, are significantly higher than those in normal-weight individuals. Their expression levels also correlate positively with body mass index (BMI) and visceral fat area [105,106]. However, the correlations with BMI are modest, typically in the weak-to-moderate range, with reported correlation coefficients of approximately 0.43 for CRP and 0.3 to 0.4 for IL-6 [107,108]. Moreover, there is substantial overlap in cytokine levels between obese and lean individuals. This suggests that the inflammatory response to obesity is not uniform: some obese individuals exhibit robust inflammation, while others maintain relatively normal cytokine levels. The determinants of this inter-individual variability, whether genetic, environmental, or microbial, are not fully understood but may underlie the distinction between metabolically healthy and unhealthy obesity [109]. HFD-induced obese models recapitulate this inflammatory signature, displaying robust M1 macrophage infiltration in VAT and marked transcriptional upregulation of mRNA expression levels of TNF-α, IL-6, and other pro-inflammatory cytokines, consistent with human clinical observations [110].

#### 2.2.2. Core Molecular Mechanisms of Inflammation-Driven Oxidative Stress

Inflammation drives oxidative stress through multiple interconnected molecular mechanisms, among which NOX family-mediated ROS generation is the most central pathway. These mechanisms involve the interplay of NOX activation, lipid mediators, mitochondrial ROS, oxidized phospholipids (OxPLs), and gut microbiota dysbiosis. Together, they form a complex regulatory network. Distinct experimental evidence from cellular, animal, and human studies collectively supports these layered pathogenic pathways. At the molecular level, excessive ROS production in obese AT is closely associated with specific upregulation of the NOX system. In WAT of KKAy obese mice, mRNA expression levels of NOX subunits are significantly elevated. These subunits include gp91^phox, which is also known as NOX2, as well as p22^phox, p47^phox, p67^phox, and p40^phox. Expression of its transcriptional regulator PU.1 is also increased. No such changes are observed in the liver or skeletal muscle [90]. Human evidence for adipose NOX upregulation remains limited to circulating plasma subunit quantification or limited biopsy analyses from cardiac surgery cohorts, with no tissue-specific transcriptional or functional data from obese patient biopsies available to validate preclinical observations. NOX4 is the most extensively characterized NOX isoform in adipocytes, exerting opposing protective and pathogenic effects that are determined entirely by the metabolic context. Under physiological lean conditions, NOX4-derived H_2_O_2_ mediates beneficial metabolic signaling: H_2_O_2_ inhibits protein tyrosine phosphatase PTP1B to preserve intact insulin signal transduction and activates MAP kinase phosphatase-1 (MKP-1) to support preadipocyte differentiation and healthy AT expansion [111,112]. In contrast, chronic exposure to excess saturated fatty acids (SFA) and glucose in obesity drives NOX4 transcriptional overexpression and relocalization to plasma membrane lipid rafts; aberrant membrane localization enables NOX4-derived ROS to activate NF-κB, induce monocyte chemoattractant protein-1 (MCP-1) transcription, and recruit circulating pro-inflammatory monocytes into AT stroma [113]. The pathophysiological relevance of these cell-autonomous effects is further supported by genetic studies: global NOX4 knockout unexpectedly accelerates HFD-induced IR, an effect attributed to impaired adipogenic differentiation and limited AT expandability, whereas adipocyte-specific NOX4 deletion improves systemic insulin sensitivity by restricting local ROS-mediated inflammatory signaling [113,114]. Collectively, these findings indicate that NOX4 plays context-dependent roles, with beneficial effects in healthy adipocytes and detrimental effects under obese conditions, and that its functions are not cell-autonomous but involve complex crosstalk between adipocytes and immune cells. The context-dependent nature of NOX4 action underscores a broader principle: the functional outcome of oxidative stress pathways depends critically on cell type, subcellular localization, and the presence of concurrent metabolic stressors. Beyond AT, NOX-mediated ROS production in vascular endothelial cells and renal tissue scavenges bioavailable nitric oxide, forming peroxynitrite and damaging vascular wall architecture, thereby driving endothelial dysfunction, a core pathogenic precursor of obesity-related hypertension and cardiovascular complications [115,116].

SFA, including palmitate and stearate, that are released from hypertrophic adipocytes act as endogenous TLR4 ligands, activating downstream pro-inflammatory cascades and NOX-dependent ROS generation in both adipocytes and tissue-resident macrophages [117,118]. HFD-fed TLR4-deficient mice exhibit attenuated macrophage infiltration, NF-κB and MCP-1 expression, and improved insulin sensitivity [29]. These genetic studies provide evidence that TLR4 signaling is causally involved in obesity-associated inflammation. However, they do not establish whether the critical TLR4 signaling occurs in adipocytes, macrophages, or both, and they do not address whether the relevant TLR4 ligands are SFAs, LPS from gut microbiota, or both. The interpretation is further complicated by the fact that TLR4-deficient mice exhibit developmental alterations that may affect their metabolic phenotype independently of adult TLR4 signaling. TLR4-dependent palmitate signaling activates NOX enzymes to boost intracellular ROS levels, upregulates pro-inflammatory cytokines IL-1β, IL-6, and TNF-α, and disrupts insulin transduction via serine phosphorylation of IRS-1 at residue Ser307 [119]. The concept of serine phosphorylation as a mechanism of IR has been challenged by recent evidence suggesting that serine phosphorylation may be a marker rather than a mediator of IR and that its functional significance may be context-dependent. Bidirectional cross-talk exists between mitochondrial ROS and inflammation. Mitochondrial ROS directly activate the IKK complex by oxidizing the NF-κB essential modulator, thereby linking impaired mitochondrial respiration to NF-κB-driven inflammation [120], whereas ROS-triggered mitochondrial damage activates the NLRP3 inflammasome, a critical checkpoint for IL-1β maturation in macrophages [121]. However, most studies demonstrating this crosstalk have employed pharmacological agents or genetic manipulations that produce acute, supraphysiological ROS bursts, whereas the chronically elevated but moderate ROS levels characteristic of obesity may engage different signaling thresholds. Conversely, pro-inflammatory factors such as TNF-α and IL-6 further disrupt mitochondrial structural integrity and ETC function, reinforcing a feedforward pathological loop of concurrent mitochondrial dysfunction and inflammation.

Extending from this mitochondria-initiated inflammatory cascade, a mutual antagonistic interaction exists between NRF2 and NF-κB at the transcriptional level. The NF-κB p65 subunit suppresses NRF2-ARE signaling by competing with NRF2 for the transcriptional co-activator CBP while promoting recruitment of histone deacetylase 3(HDAC3) to MAF bZIP transcription factor K (MafK) and by directly interacting with Keap1 to enhance NRF2 ubiquitination and degradation [122,123]. Conversely, NRF2 negatively regulates NF-κB signaling by increasing antioxidant defenses and inducing HO-1 expression [124,125,126]. The regulation of the NLRP3 inflammasome by NRF2 is context-dependent. Some studies have reported that NRF2 is essential for activating the NLRP3 and AIM2 inflammasomes [127], whereas others have demonstrated that NRF2 inhibits NLRP3 inflammasome activity by reducing ROS-induced NLRP3 priming, thereby decreasing caspase-1 cleavage and IL-1β production [128]. These discrepancies likely arise from differences in experimental models, the nature of the inflammatory stimulus, and the timing of NRF2 activation. Genetic ablation of NRF2 may compromise cellular stress adaptation required for inflammasome assembly, whereas pharmacological activation engages an antioxidant program that directly counteracts ROS-driven NLRP3 priming. Of particular relevance to obesity, ROS act as a double-edged signal given that moderate levels promote NLRP3 transcription and inflammasome activation, whereas sustained oxidative stress in the obese AT suppresses caspase-1 via reversible oxidation of its redox-sensitive cysteine residues. In this chronic low-grade inflammatory context, NRF2 activation may serve as a feedback restraint, limiting excessive inflammasome activity and maintaining redox homeostasis, in contrast to its pro-inflammatory role in acute host defense. This discrepancy reflects the complexity of NRF2’s role in inflammatory regulation, which varies by cell type and stress conditions. The field would benefit from standardized experimental protocols that systematically vary the timing, duration, and intensity of NRF2 activation to clarify these context-dependent effects.

OxPLs represent an additional redox-dependent signaling intermediate linking oxidative stress and macrophage polarization. In the stromal vascular fraction of epididymal WAT from obese mice, the ratio of full-length to truncated oxidized phosphatidylcholine is significantly elevated relative to lean littermates [29]. Truncated OxPL species preferentially induce antioxidant gene transcription, whereas full-length OxPLs drive a pro-inflammatory transcriptional signature including IL-1β, IL-6, and CXCL-1 that polarizes resident adipose macrophages toward the M1 phenotype [129]. To date, no human AT OxPL lipidomic profiling studies have validated this polarization mechanism in obese patient samples, and the lipidomic methods required for OxPL quantification are technically demanding and not widely standardized. Gut microbiota dysbiosis represents another important source of obesity-associated oxidative stress. The gut microbiota is the most diverse microbial community in the human body. It plays a critical role in maintaining nutrient metabolism, energy uptake, epithelial homeostasis, and immune function. Unhealthy lifestyle factors such as HFD and antibiotic use can lead to dysbiosis [130,131]. This dysbiosis increases intestinal permeability and triggers metabolic endotoxemia as well as chronic low-grade systemic inflammation. Metabolic endotoxemia is characterized by increased lipopolysaccharide (LPS) [132,133]. These events in turn promote ROS production and oxidative stress. Circulating LPS penetrates peripheral metabolic tissues, stimulating widespread NOX-dependent ROS generation and chronic low-grade inflammatory signaling, which synergistically exacerbate obesity-related metabolic dysfunction. A critical limitation of the gut microbiota literature is that nearly all mechanistic studies rely on germ-free or antibiotic-treated mouse models. While these models have provided proof of concept for microbiota-immune interactions, they are fundamentally different from human obesity, where microbiota composition shifts gradually over years rather than being acutely removed or transplanted. Human fecal microbiota transplant intervention trials are limited and lack tissue-specific redox biomarker readouts to confirm translational relevance. Moreover, the specific bacterial taxa and metabolites responsible for ROS generation remain incompletely characterized, and the extent to which microbiota-derived signals contribute to oxidative stress relative to host-derived signals has not been quantified. In summary, the molecular mechanisms linking inflammation to oxidative stress in obesity are characterized by a high degree of contextual complexity. Pathways that appear protective in one context may be pathogenic in another, and the translation of preclinical findings to human obesity requires careful attention to cell type, experimental model, and metabolic context.

#### 2.2.3. Placental Inflammation and Systemic Impact

Obesity-driven systemic inflammatory signaling also disrupts placental tissue homeostasis during pregnancy. Maternal obesity correlates strongly with high-grade chronic villitis, an inflammatory placental lesion linked to adverse gestational outcomes, including fetal growth restriction and stillbirth; obese pregnancies additionally display elevated trophoblastic TNF-α expression and abnormal placental morphological remodeling [134,135,136]. While the association between maternal obesity and placental inflammation is consistently reported, the specific inflammatory phenotype, particularly macrophage density and cytokine expression, has been the subject of contradictory findings. Some studies have reported increased placental macrophage density, particularly in male fetuses of obese women [137]. Other studies have observed decreased macrophage populations and reduced cytokine signaling [138]. These conflicting findings suggest that the specific inflammatory phenotype may depend on maternal obesity severity, study population, and experimental approaches. Potential contributing factors include differences in BMI classification thresholds, gestational age at sampling, the presence of metabolic complications such as gestational diabetes, the choice of macrophage markers, including CD68, CD163, or HLA-DR, tissue sampling location, whether from placental disc, membranes, or decidua basalis, and statistical power to detect fetal sex-specific effects. Importantly, placental studies are limited by small sample sizes.

Collectively, inflammation and oxidative stress are intimately linked in obesity. Inflammatory cytokines induce ROS production via activation of NOX and iNOS, while ROS activate redox-sensitive transcription factors such as NF-κB, further enhancing inflammatory mediator expression. This bidirectional relationship establishes a vicious cycle in which inflammation induces oxidative stress, which in turn induces further inflammation, thereby accelerating obesity-associated metabolic dysfunction. This axis is closely intertwined with mitochondrial dysfunction, ER stress, and dysregulation of the antioxidant system, collectively forming the core molecular network of obesity-associated oxidative stress. In summary, the evidence for placental inflammation in maternal obesity is consistent, but the specific phenotype is heterogeneous across studies, and the causality and clinical significance of these changes remain unresolved. Methodological standardization and larger, well-controlled studies are needed to clarify these uncertainties.

### 2.3. ER Stress and Oxidative Stress Crosstalk

The ER mediates the synthesis, folding, and post-translational modification of all secreted and membrane-bound proteins, as well as regulated intracellular calcium storage and release [139]. In obesity, sustained excess nutrient influx overwhelms the ER’s protein-folding capacity, leading to the accumulation of misfolded proteins and activation of the unfolded protein response (UPR) [140]. In the liver of obese models, altered ER membrane lipid composition and severely impaired ER calcium retention capacity are observed, further exacerbating ER functional failure [141]. The UPR signaling cascade is coordinated by three canonical ER-resident sensor proteins: IRE1, PERK, and ATF6, which collectively remodel cellular transcriptional and translational programs to restore basal ER homeostasis under acute stress conditions [142].

#### 2.3.1. Bidirectional Causal Relationship Between ER Stress and Oxidative Stress

**Figure 2:**
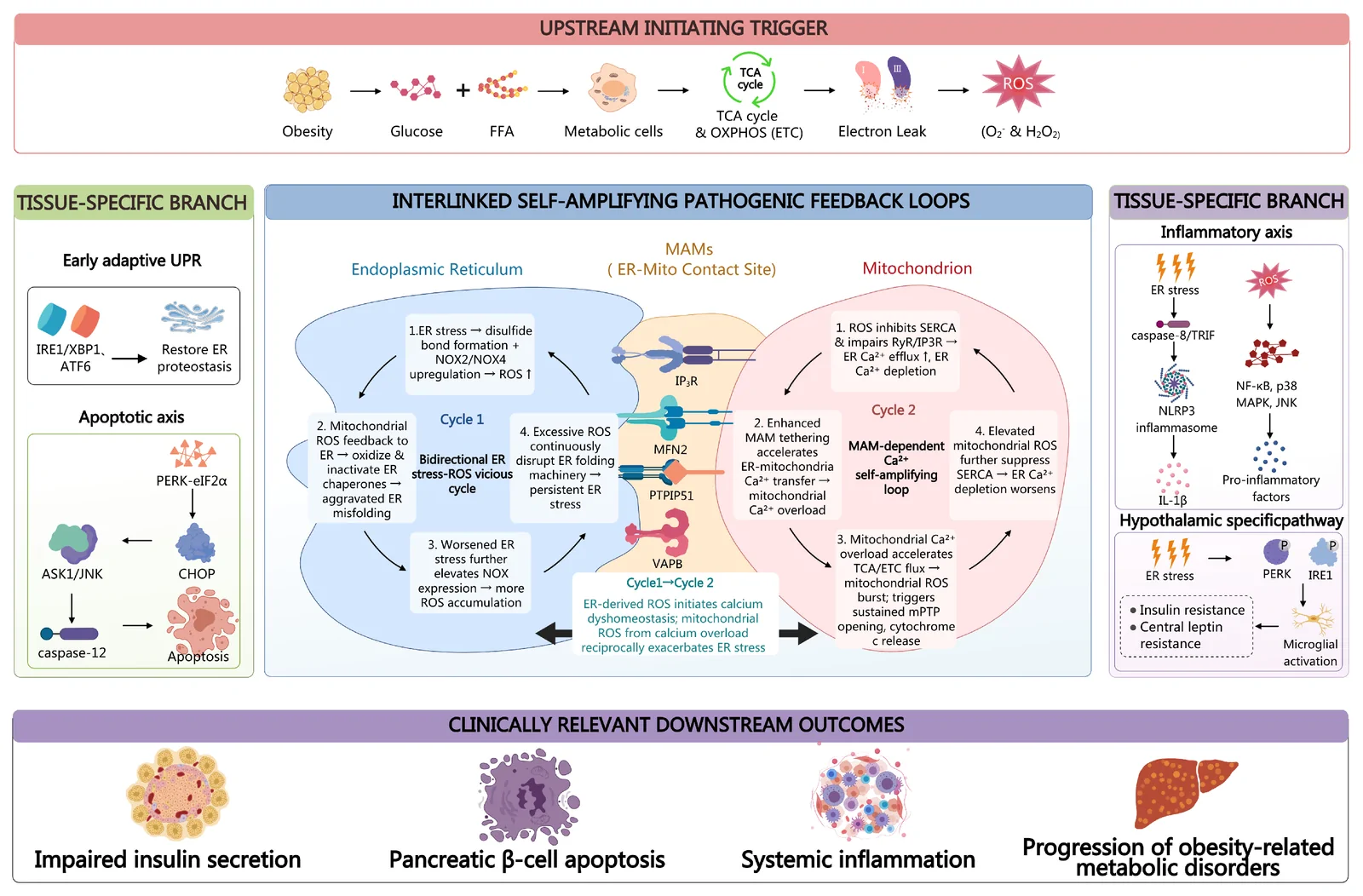
Crosstalk between ER stress and oxidative stress in obesity.

A bidirectional causal relationship exists between ER stress and oxidative stress [143]. Disrupted ER protein folding machinery impairs disulfide bond formation and intracellular electron equilibrium, driving uncontrolled ROS release and widespread cytosolic redox imbalance [144,145]. Conversely, surplus ROS originating from mitochondrial leakage, inflammatory NOX activation, or chronic nutrient overload directly oxidizes ER chaperone proteins and redox-sensitive cysteine residues, disrupting native protein folding and sustaining persistent pathological ER stress [146]. ROS modulate ER calcium handling through two complementary regulatory mechanisms: ROS impair the gating function of ryanodine receptors (RyR) and inositol 1,4,5-trisphosphate receptors (IP3R), promoting unregulated calcium efflux from the ER lumen into the cytosol and mitochondrial matrix, while simultaneously suppressing sarco-ER Ca^2+^-ATPase (SERCA) catalytic activity to block ER calcium reuptake, permanently destabilizing intracellular calcium homeostasis [147]. Calcium dysregulation further impairs protein maturation and secretory trafficking, establishing a feedforward pathological loop linking ER structural malfunction and progressive oxidative biomolecular damage. The three canonical UPR branches—IRE1, ATF6, and PERK—not only resolve protein misfolding but also modulate ROS production [148]. Prolonged unresolved ER stress rewires UPR transcriptional output to upregulate NOX2 and NOX4 isoforms, stimulating supplementary ROS generation required for ER membrane remodeling and proteasomal clearance of misfolded proteins. However, the temporal sequence of events, specifically whether NOX upregulation is a cause or a consequence of persistent ER stress, has not been firmly established, as most studies have examined only single time points. Sustained excessive ROS accumulation eventually overwhelms endogenous antioxidant capacity and exacerbates global redox imbalance and cellular injury [149,150,151]. In summary, the bidirectional relationship between ER stress and oxidative stress is strongly supported by evidence, but the mechanistic details, particularly the relative contributions of the three UPR branches and the temporal sequence of events, remain incompletely characterized. The relevance of pharmacological ER stress models to chronic obesity-associated ER stress requires further validation. The interplay among ER stress, oxidative stress, calcium dysregulation, and downstream inflammatory signaling is shown in Figure 2.

Excess glucose and FFA overload the ER folding capacity, accumulate unfolded proteins, and activate three canonical UPR axes (IRE1, PERK, and ATF6). Two synergistic feedforward loops dominate core pathogenesis: (1) Bidirectional ER stress-ROS loop: ER stress elevates ROS via disulfide bond synthesis and NOX2/NOX4 upregulation; mitochondrial ROS oxidizes ER chaperones to worsen protein misfolding reciprocally. (2) MAM calcium overload loop: ROS disturbs RyR/IP3R and represses SERCA to trigger ER Ca^2+^ leakage; strengthened MAM contacts facilitate ER-mitochondria Ca^2+^ transfer to induce mitochondrial Ca^2+^ overload, whose elicited ROS further suppresses SERCA and depletes ER Ca^2+^ stores. UPR exerts stage-dependent effects: early adaptive IRE1/XBP1 and ATF6 signaling restores ER proteostasis, whereas sustained stress shifts UPR toward pro-apoptotic CHOP, ASK1/JNK, and caspase-12 cascades. ER stress initiates the NLRP3 inflammasome through caspase-8/TRIF for IL-1β release, and ROS activates NF-κB/p38 MAPK/JNK to stimulate pro-inflammatory cytokine production. An independent hypothalamic module shows that ER stress increases PERK/IRE1 phosphorylation and microglial activation, thereby causing central leptin resistance and IR, which disrupts energy homeostasis. Collectively, these signaling cascades impair insulin secretion and induce β-cell apoptosis and systemic inflammation, thereby facilitating the progression of obesity-related metabolic diseases. (ER, endoplasmic reticulum; FFA, free fatty acids; UPR, unfolded protein response; ROS, reactive oxygen species; NOX, NADPH oxidase; MAMs, mitochondria-associated ER membranes; RyR, ryanodine receptor; IP3R, inositol 1,4,5-trisphosphate receptor; SERCA, sarco-ER Ca^2+^-ATPase; TCA, tricarboxylic acid; ETC, electron transport chain; mPTP, mitochondrial permeability transition pore; CHOP, C/EBP homologous protein; ASK1, apoptosis signal-regulating kinase 1; JNK, c-Jun N-terminal kinase; NLRP3, nucleotide-binding oligomerization domain-like receptor protein 3; IL-1β, interleukin-1β; NF-κB, nuclear factor κB; MAPK, mitogen-activated protein kinase; IR, insulin resistance).

#### 2.3.2. ER-Mitochondrial Communication and Calcium Dyshomeostasis in Obesity

Physical and functional coupling between the ER and mitochondria occurs at specialized subcellular domains termed MAMs, which maintain the two organelles at a narrow distance of 10–30 nm to enable rapid transfer of calcium, lipids, and signaling molecules [152,153]. MAMs are highly enriched in structural and regulatory proteins, including IP3R, MFN2, PACS-2, and Sigma-1 receptor (Sig1R), which collectively stabilize contact formation and mediate signal transmission [154,155,156]. The ER-resident vesicle-associated membrane protein VAPB directly interacts with the outer mitochondrial membrane protein PTPIP51, forming a tethering complex that governs inter-organelle proximity and signaling efficiency [157]. Notably, obesity markedly potentiates ER-mitochondrial contact formation in metabolic tissues such as the liver. Electron microscopy and quantitative imaging of ob/ob and HFD-induced obese models reveal expanded ER-mitochondria juxtaposition relative to lean controls [158,159]. This structural remodeling is accompanied by elevated expression of core MAM components, including IP3R1, IP3R2, PACS-2, and Sig1R [160,161]. Forced overexpression of VAPB or PTPIP51 amplifies ER-mitochondrial contact, triggering mitochondrial calcium overload, mitochondrial permeability transition pore (mPTP) opening, cytochrome c release, and ROS burst, ultimately promoting apoptotic cell death [157]. Synthetic pharmacological enhancement of ER-mitochondrial contact exacerbates hepatic steatosis and systemic glucose intolerance in obese mice, whereas genetic knockdown of IP3R1 or PACS-2 normalizes pathological calcium flux, restores mitochondrial oxidative function, alleviates cellular stress, and improves whole-body glucose tolerance [141,162,163]. Human translational data remain limited to indirect quantification of mRNA expression from liver biopsies of obese patients, with no direct ultrastructural MAM imaging or gene-manipulation studies performed in human primary metabolic tissues.

Enhanced MAM coupling drives excessive unregulated calcium translocation from the ER lumen into the mitochondrial matrix. Hepatocytes isolated from obese animals display elevated basal mitochondrial calcium concentrations and exaggerated calcium uptake kinetics following ER calcium release [141,160,161]. Mitochondrial calcium overload exerts multiple pathogenic effects: it stimulates TCA cycle dehydrogenases and accelerates ETC flux, thereby increasing ROS production; it promotes sustained mPTP opening, leading to membrane depolarization, organelle swelling, and the release of pro-apoptotic factors; and it sensitizes ER calcium release via ROS-dependent mechanisms, thereby reinforcing a vicious cycle of ER and mitochondrial damage [147]. A major translational limitation of current MAM research is that functional genetic and pharmacological evidence remains almost exclusively derived from rodent hepatic models. Whether comparable pathological MAM expansion and calcium dysregulation occur in human obese AT, skeletal muscle, or pancreatic β-cells remains entirely uncharacterized, as human data are largely confined to in vitro cultured cell systems rather than patient biopsy specimens.

#### 2.3.3. Downstream Signaling Pathways of ER Stress and Cell Fate

ER stress modulates autophagic and apoptotic signaling to determine cell fate under obese conditions. The PERK/eIF2α axis upregulates multiple autophagy-related genes, including ATG4, ATG5, and ATG12 [164,165]. EIF2α-mediated transcriptional induction of CHOP disrupts the inhibitory Beclin1-Bcl2 protein complex, further amplifying adaptive autophagic flux to resolve ER protein overload. The functional significance of CHOP in human obesity is further complicated by the existence of multiple isoforms that may exert distinct or even opposing functions. The DDIT3/CHOP gene gives rise to several alternatively spliced transcripts, encoding at least two protein isoforms of differing lengths. Notably, one study demonstrated that cytoplasmic and nuclear-localized DDIT3 regulate largely non-overlapping gene expression profiles: among 175 identified DDIT3 target genes, only three were regulated by DDIT3 in both subcellular compartments [166]. However, experimental studies in cell and animal models rarely distinguish between these isoforms, typically treating CHOP as a single factor, as exemplified by CHOP knockout mice. This limitation hampers efforts to delineate the specific role of CHOP in human AT, adipogenesis, and the inflammatory aspects of obesity. Despite this complexity, under severe, unresolved long-term ER stress, UPR signaling switches from adaptive protective programs to pro-apoptotic cascades via three independent core axes: transcriptional activation of CHOP/GADD153, stimulation of the ASK1/JNK kinase cascade, and induction of caspase-12-dependent apoptotic signaling [167,168,169]. ER stress also promotes macrophage IL-1β maturation and secretion via caspase-8- and TRIF-dependent pathways following TLR4 ligation, thereby directly linking ER stress to sterile inflammatory activation and NLRP3 inflammasome assembly [170,171]. Metabolic stressors, including LPS, palmitate, and oleate, synergize with ER stress to amplify inflammatory responses. In monocytic models, combined stress induces TNF-α expression through ROS/CHOP/HIF-1α and MAPK/NF-κB signaling modules, which are further enhanced by oxidative insults [172,173,174,175,176]. Notably, pretreatment with antioxidants such as curcumin, apocynin, or allopurinol significantly attenuates stress-induced TNF-α production, supporting the therapeutic potential of redox modulation in mitigating ER stress-related inflammation. These observations support the therapeutic potential of redox modulation in mitigating ER stress-related inflammation. However, the antioxidant concentrations required for efficacy in vitro are often substantially higher than those achievable in vivo, and the specificity of these agents at lower concentrations remains uncertain. As critical second messengers, ROS activate an array of signaling cascades, including PI3K/Akt, p38 MAPK, ERK, JNK, PKC, and Src-family kinases, all of which converge to promote pro-inflammatory cytokine expression [147,177]. Moreover, ROS stimulates redox-sensitive transcription factors, including NF-κB, NRF2, and HIF-1α, thereby propagating and sustaining inflammatory signaling in metabolic tissues [178].

#### 2.3.4. Central ER Stress and Energy Balance Regulation

Beyond peripheral metabolic tissues, ER stress occurs in the central nervous system, where it contributes to the pathogenesis of obesity by disrupting energy balance. In both dietary and genetic models of obesity, hypothalamic ER stress is robustly induced, as evidenced by elevated phosphorylation of PERK and IRE1, alongside microglial activation and increased production of pro-inflammatory cytokines such as IL-6, TNF-α, and IL-1β [179,180,181]. Central administration of the ER stressor thapsigargin impairs the anorexigenic actions of leptin and insulin, indicating that hypothalamic ER stress promotes central leptin and IR [182]. Conversely, chemical chaperones such as 4-phenylbutyric acid (4-PBA) and tauroursodeoxycholic acid (TUDCA) reduce body weight and food intake, enhance leptin sensitivity, and elevate energy expenditure in obese mice. In line with these observations, constitutive XBP1 expression in hypothalamic pro-opiomelanocortin (POMC) neurons confers resistance to HFD-induced obesity in vivo [181]. Collectively, these findings provide compelling evidence for a causal role of hypothalamic ER stress in energy balance dysregulation. Nevertheless, several limitations warrant consideration. Chemical chaperones exert broad effects on protein folding throughout the body, and their specificity for hypothalamic ER stress has yet to be established [183]. Consequently, the observed metabolic benefits may be partially attributable to peripheral actions on the liver or AT rather than direct central effects. Furthermore, the doses used in rodent studies are often substantially higher than those considered feasible or safe in humans [184]. Although genetic manipulation of XBP1 in POMC neurons offers stronger evidence for a causal role of central ER stress, its relevance to human obesity remains unclear, given that XBP1 splicing and other UPR markers have not been systematically examined in postmortem hypothalamic tissue from obese individuals. Nonetheless, the available data indicate that central ER stress disrupts hypothalamic nutrient sensing and hormone signaling, thereby promoting hyperphagia, reducing energy expenditure, and contributing to obesity progression.

In summary, while the evidence supporting a role for hypothalamic ER stress in obesity is robust in rodent models, translation to human disease remains limited by the inherent challenges of assessing central ER stress in living individuals and the scarcity of postmortem tissue studies. Future investigations should employ neuroimaging approaches or cerebrospinal fluid biomarkers to evaluate hypothalamic ER stress in human obesity and to determine the translational potential of targeting this pathway.

### 2.4. Dysregulation of Endogenous Antioxidant Defense Systems

#### 2.4.1. Components of the Antioxidant Defense System

Under physiological conditions, mammalian cells maintain intracellular redox homeostasis via a sophisticated, hierarchically coordinated antioxidant defense network composed of enzymatic and non-enzymatic components, which jointly counteract ROS overaccumulation and sustain cellular metabolic stability. Non-enzymatic antioxidants comprise dietary nutrients such as vitamin C, vitamin E, carotenoids, and polyphenols, as well as endogenously synthesized molecules including uric acid and glutathione (GSH). These compounds act as the first-line redox buffer, directly neutralizing excess ROS and mitigating initial oxidative damage to biomolecules, with broad-spectrum protective effects across multiple cell types [185].

The enzymatic antioxidant system consists of three core functionally coupled cascades with distinct subcellular localization and catalytic specificity. Superoxide dismutases (SODs), classified into Cu/Zn-SOD (cytosolic), Mn-SOD (mitochondrial), Fe-SOD, and Ni-SOD isoforms based on metal prosthetic groups, specifically catalyze the dismutation of O_2_^−^ into H_2_O_2_ [186]. Subsequently, catalase (CAT) and glutathione peroxidases (GPx) detoxify cytotoxic H_2_O_2_ into inert water, while the thioredoxin (Trx) system, consisting of thioredoxin reductase, NADPH, and Trx, maintains protein thiol redox balance, modulates redox-dependent signaling, and supports secondary ROS clearance via 2-cysteine peroxidases [57,187,188]. The Trx system continuously regenerates reduced Trx to sustain long-term antioxidant, anti-apoptotic, and transcriptional regulatory functions [188]. Complementarily, the glutathione system includes GPx1, GPx4, and GSH, whose synthesis from glutamate, cysteine, and glycine is tightly regulated by glutamate-cysteine ligase and glutathione synthase. GPx-mediated catalysis requires reduced GSH as an electron donor, and the GSH/GSSG ratio is widely recognized as a core indicator of cellular redox balance [189,190,191]. Collectively, these interconnected pathways preserve mitochondrial structural integrity and systemic redox stability under physiological conditions, laying the foundation for intact metabolic function. The functional interdependence among these pathways has important implications for obesity research: a deficiency in one component, such as GSH depletion due to impaired synthesis or reduced NADPH regeneration, can compromise the entire system. This systems-level perspective is essential for interpreting the often heterogeneous findings regarding individual antioxidant enzymes in obese populations, as the functional impact of a deficiency in any single component depends critically on the reserve capacity of the other components.

#### 2.4.2. Impaired Antioxidant Enzyme Activity and Glutathione Depletion in Obesity

In obesity, the endogenous antioxidant defense system undergoes progressive and systemic functional collapse across metabolically active tissues, including AT, liver, and skeletal muscle. Numerous clinical and preclinical studies consistently demonstrate reduced catalytic activity and protein expression of SOD, CAT, and GPx in obese models and individuals, directly correlating with elevated systemic oxidative stress [192,193]. Mitochondria, the primary cellular ROS production site, possess a specialized mitochondrial antioxidant panel (SOD2, Trx2, TrxR2, Prx3) to limit ROS leakage during oxidative phosphorylation. Preclinical studies confirm that obesity markedly inhibits the expression and activity of these mitochondrial-specific antioxidants, inducing mitochondrial oxidative damage and further amplifying ROS generation, thus forming a pro-oxidative positive feedback loop [194,195]. Moreover, animal experiments verify that forced overexpression of mitochondrial antioxidant enzymes effectively rescues HFD-induced IR, establishing a causal link between mitochondrial antioxidant deficiency and metabolic dysfunction in preclinical settings [196]. In vitro studies have established that palmitate-induced lipotoxicity triggers GSH depletion in metabolic cells, including human hepatocytes, primary rat hepatocytes, and pancreatic β-cells [197]. These findings provide mechanistic insight into how excess fatty acids directly compromise antioxidant capacity at the cellular level, independent of systemic factors. However, the supraphysiological palmitate concentrations used in many such studies warrant caution in extrapolating these findings to human physiology. Human clinical evidence is largely observational and less conclusive compared with preclinical data. Clinical cohort studies demonstrate that patients with obesity and metabolic syndrome exhibit significantly lower plasma SOD/CAT/GPx activity, a higher GSSG/GSH ratio, and elevated oxidative damage biomarkers (MDA, 4-HNE, protein carbonyls, 8-OHdG) than healthy individuals, indicating a strong correlation between antioxidant deficiency and human obesity-related oxidative stress [198,199,200,201,202,203,204,205]. These observations establish a clear association between obesity and compromised antioxidant status in humans, but the nature of this association remains fundamentally unclear. The cross-sectional design of these studies cannot distinguish whether reduced antioxidant enzyme activity precedes obesity and contributes to its development or whether it emerges as a consequence of chronic oxidative stress. Additionally, clinical observations confirm that obesity-associated hypomagnesemia further impairs antioxidant function by inhibiting magnesium-dependent antioxidant enzymes and promoting inflammatory ROS production; pediatric obesity studies specifically verify that reduced serum magnesium is positively correlated with plasma MDA levels in human subjects [23,206,207,208,209,210]. Several methodological considerations further complicate the interpretation of human studies. Plasma enzyme activities may not reflect tissue-specific antioxidant capacity, as erythrocyte SOD and GPx can be influenced by hematological parameters that differ between obese and lean individuals. For example, mean corpuscular volume (MCV) is lower in overweight and obese children than in normal-weight controls, whereas red cell distribution width (RDW) is elevated in obesity and is associated with inflammatory markers [211,212]. Assays for oxidative damage biomarkers, particularly MDA and protein carbonyls, are susceptible to ex vivo artifacts during sample processing, including hemolysis, delayed centrifugation, and repeated freeze–thaw cycles. It remains unclear whether antioxidant system dysfunction is a primary driver of human obesity pathogenesis or a secondary compensatory consequence of long-term lipid accumulation and chronic inflammation, representing a critical unresolved question in this field. Current studies mostly focus on the overall inhibitory effect of obesity on antioxidant systems, while controversies remain regarding tissue-specific sensitivity. Some studies suggest that hepatic antioxidant enzymes are more vulnerable to lipotoxic damage in obesity, whereas subcutaneous AT may retain a partial compensatory antioxidant capacity in the early stages of obesity. This tissue-specific heterogeneity is biologically plausible given the different metabolic demands and lipid exposure of various tissues, but no unified conclusion has been reached due to differences in patient age, BMI stratification, and disease duration among obese cohorts.

#### 2.4.3. Pathological Suppression of the NRF2/Keap1 Pathway and Antioxidant Transcriptional Failure

The NRF2/Keap1 signaling axis functions as the master transcriptional regulator of cellular adaptive antioxidant responses [213]. Under basal conditions, Keap1 constitutively binds NRF2 and promotes its ubiquitination and proteasomal degradation to maintain low baseline activity. During oxidative stress, ROS-mediated oxidation of Keap1 disrupts its repressive function, allowing NRF2 to translocate into the nucleus and drive the transcription of antioxidant genes [214]. Upon nuclear translocation, NRF2 heterodimerizes with small Maf proteins and binds to antioxidant response elements (AREs), thereby upregulating a cohort of cytoprotective genes, including SOD1, CAT, GPx, HO-1, NQO1, and GCLC, the rate-limiting enzyme for GSH synthesis [215,216,217]. This pathway represents a critical adaptive mechanism that couples oxidative challenge to transcriptional activation of antioxidant defenses. In the context of obesity, however, the protective capacity of this system is compromised. The status of NRF2 in obesity is complex and appears to be tissue-specific. Accumulating evidence indicates that in key metabolic tissues such as the liver, NRF2 transcriptional activity is suppressed, leading to reduced expression of its downstream antioxidant targets [218]. In AT, however, findings are inconsistent, with some studies reporting NRF2 activation as a compensatory response insufficient to fully counteract oxidative stress, while others observe suppression of its nuclear translocation and transcriptional activity [219]. Specific NRF2 knockdown models have revealed that NRF2 loss can attenuate HFD-induced obesity and inflammation via inhibition of the cGAS-STING pathway [220], consistent with systemic NRF2 knockout studies showing increased energy expenditure and resistance to HFD-induced obesity, mediated in part by FGF21 upregulation and WAT UCP1 elevation [221,222]. However, the lack of depot- and cell-type specificity in most studies, together with evidence that adipocyte-specific NRF2 deletion can worsen IR in some contexts, underscores that the NRF2 response to obesity is not uniform and may be modulated by local factors including inflammation, tissue metabolic activity, and depot location. Notably, the inactivation of NRF2 in obesity is not merely a passive consequence of excessive ROS production but an active inhibitory process driven by chronic inflammatory signaling. Pro-inflammatory cytokines, particularly TNF-α and IL-1β, directly impair NRF2 nuclear translocation and reduce its transcriptional activity, establishing a vicious cycle whereby inflammation suppresses antioxidant defense and oxidative stress in turn amplifies inflammation [223,224]. This pathogenic crosstalk abolishes the adaptive antioxidant response and locks cells in a state of irreversible redox imbalance.

In addition to its established role in coordinating glutathione-dependent antioxidant defenses, NRF2 directly orchestrates a multi-layered transcriptional program to suppress ferroptosis, a distinct form of iron-dependent cell death driven by catastrophic lipid peroxidation [225,226]. Specifically, NRF2 transcriptionally activates SLC7A11 to sustain glutathione biosynthesis [227], upregulates ferritin heavy and light chains (FTH1/FTL) and ferroportin (FPN1/SLC40A1) to restrict the labile iron pool [228,229,230,231,232], and induces ferroptosis suppressor protein 1 (FSP1/AIFM2) to maintain the pool of the lipophilic radical-trapping antioxidant CoQH_2_ through regeneration from its oxidized form CoQ10 [233,234,235,236]. These NRF2-dependent ferroptosis defense mechanisms are of particular relevance to obesity-related metabolic complications [237,238,239,240,241,242,243]. The concept of NRF2-regulated ferroptosis defense is mechanistically compelling and has generated considerable interest, but several methodological and interpretative challenges remain. The specificity of ferroptosis markers, particularly GPx4 and ACSL4, has been questioned because these proteins also participate in other cellular processes [244]. The concentration of labile iron, a critical determinant of ferroptosis susceptibility, is difficult to measure accurately in intact tissues. Moreover, the existence and functional significance of ferroptosis in human obesity have not been directly demonstrated; most evidence comes from rodent models and cell culture systems. The assays used to detect ferroptosis in tissues, such as malondialdehyde and 4-HNE measurement, are not specific to ferroptosis and are elevated in other forms of cell death and oxidative stress. Despite these limitations, the concept of ferroptosis as a mediator of obesity-associated tissue damage opens new avenues for therapeutic intervention, although translation to clinical applications remains distant.

#### 2.4.4. Tissue-Specific Dysregulation and Compensatory Antioxidant Responses

Obesity-related antioxidant disruption shows clear tissue-specific patterns, especially in metabolically active and stress-sensitive organs such as the placenta. In maternal obesity, term placentas exhibit significantly reduced total antioxidant capacity and catalase activity, accompanied by elevated iNOS expression and nitrative stress, indicating an adaptive but overwhelmed antioxidant response [245]. Moreover, decreased physical activity and increased sedentary behavior during pregnancy further elevate placental oxidative stress markers, confirming that lifestyle directly modulates tissue-specific redox vulnerability [246]. Beyond tissue-specific changes, cells can activate compensatory antioxidant programs under combined metabolic and ER stress. Studies in monocytes demonstrate that stimulation with LPS plus thapsigargin or palmitate plus thapsigargin markedly upregulates mRNA expression of SOD2 and NRF2, and that there is a strong positive correlation between the two genes (r = 0.91) [172]. This observation suggests that cells mount a coordinated transcriptional response to combined stress. However, the upregulation of SOD2 and NRF2 proteins is milder than the mRNA-level upregulation, suggesting that post-transcriptional regulation, including translation efficiency, protein stability, and degradation, critically fine-tunes antioxidant defense [247,248]. This discrepancy between mRNA and protein levels is rarely discussed in the obesity literature, yet it has important implications for interpreting transcriptomic studies that infer antioxidant capacity from gene expression alone. Furthermore, pretreatment with antioxidants such as curcumin or ROS scavengers, including apocynin, significantly suppresses stress-induced inflammatory cytokine production, supporting that enhancing endogenous antioxidant signaling represents an effective strategy to alleviate obesity-related inflammation and oxidative stress [249,250].

The NRF2 status in AT macrophages is a critical determinant of the degree of obesity-associated inflammation [251,252,253]. In M1 macrophages, NRF2 transcriptional activity is significantly suppressed by NF-κB, leading to dysregulation of the scavenger receptor CD36 and increased internalization of oxidized LDL, promoting foam cell formation. In contrast, M2 macrophages exhibit higher NRF2 activity and maintain their antioxidant phenotype by regulating HO-1 and NQO1 [251,252,253]. Therefore, restoring macrophage-specific NRF2 function may represent a key strategy to break the vicious cycle of obesity-associated inflammation.

These pathological changes across different tissues are not isolated—lipid peroxides released from AT can exacerbate oxidative burden in muscle and liver, while mitochondrial damage molecules released from the latter two propagate systemic inflammation and affect the central nervous system, forming a cross-organ feed-forward amplification loop.

The interplay between mitochondrial dysfunction, ROS signaling, and redox regulation in obesity presents several unresolved controversies that merit critical examination. Although mitochondrial dysfunction is consistently associated with obesity and IR, definitive proof of causality in humans remains limited due to the correlational nature of most cross-sectional studies and the difficulty of isolating primary mitochondrial defects from secondary metabolic derangements. The functional role of ROS in obesity is intrinsically context-dependent, as excessive ROS drives tissue damage and IR, whereas complete elimination of ROS may be detrimental by disrupting physiological redox signaling required for insulin action and immune function. The NRF2 pathway exhibits tissue- and context-specific responses to obesity, with some tissues showing suppression while others display compensatory activation. Conflicting findings regarding NOX isoforms further underscore the limitations of global genetic deletion approaches, which may obscure cell type-specific functions and elicit compensatory adaptations that confound interpretation. Collectively, these controversies highlight the need for tissue-specific targeting strategies and more sophisticated experimental models that better recapitulate the heterogeneity of human obesity.

The major molecular mechanisms of obesity-associated oxidative stress are shown in Table 1.

## 3. Biomarkers of Oxidative Stress in Obesity

Assessment of redox status in obesity largely relies on quantifying stable end-products of oxidative damage, as direct measurement of ROS remains technically challenging. Emerging evidence indicates that dysfunctional, inflamed AT acts as a major contributor to systemic oxidative stress in obesity [90]. Consequently, biomarkers that closely reflect AT-related pathological alterations are of particular clinical and mechanistic significance. The summary of oxidative stress biomarkers in obesity is shown in Table 2.

### 3.1. Validated Biomarkers

Among the numerous oxidative stress biomarkers reported in the literature, only a limited subset has been sufficiently validated for clinical application. F2-isoprostanes and 8-hydroxy-2′-deoxyguanosine (8-OHdG) represent the most robustly validated markers. F2-isoprostanes, generated via non-enzymatic peroxidation of arachidonic acid, are widely regarded as the gold standard biomarker for in vivo lipid peroxidation owing to their chemical stability and specificity [255]. Among the F2-isoprostane family, 8-epi-PGF2α has been the most extensively studied and validated in clinical settings [256]. Clinical studies have consistently demonstrated positive correlations between urinary 8-epi-PGF2α levels and BMI and waist circumference in non-diabetic individuals [90]. Animal studies further corroborate these findings, with obese KKAy mice exhibiting substantially elevated ROS production in white AT compared with lean controls [90]. Despite these advantageous analytical characteristics, the clinical utility of F2-isoprostanes is constrained by several notable limitations. Accurate quantification necessitates advanced analytical platforms such as GC-MS or LC-MS/MS, which restrict their accessibility in routine clinical practice [257]. The equipment costs and the requirement for specialized technical expertise limit implementation to specialized research laboratories. Moreover, circulating and urinary levels are susceptible to confounding influences from age [259], sex, ethnicity, and smoking status [258]. More fundamentally, inter-laboratory and inter-platform variability poses a substantial barrier to clinical translation. International ring trials on LC-MS/MS-based metabolite quantification have demonstrated that even with identical kits and standardized protocols, mean inter-laboratory CVs of approximately 8–22% are typical, with some hydrophobic analytes exceeding 30% due to matrix interference [301]. The sources of variability include differences in sample collection and handling (hemolysis, anticoagulant type, centrifugation, storage temperature, and freeze–thaw cycles), as well as instrument-specific parameters (mass resolution, ionization efficiency, and column batch effects). Although pooled QC samples improve intra-batch precision, differential CV distributions across metabolite classes reveal that internal standardization alone cannot eliminate instrument- and protocol-dependent biases. These findings highlight that without cross-platform harmonization, the clinical translation of LC-MS/MS-based biomarker panels risks being undermined by measurement uncertainty that can equal or exceed the biological effect size being studied. From an analytical sensitivity standpoint, LC-MS/MS methods achieve low pg/mL limits of detection for F2-isoprostanes, which is adequate for research purposes; however, this sensitivity has not been systematically evaluated against clinically meaningful thresholds for obesity-related metabolic risk stratification. From a cost-effectiveness perspective, per-sample analysis costs are high. Notably, obesity-specific reference ranges and validated cutoff values for metabolic risk stratification have yet to be established for this biomarker.

DNA oxidative damage represents another critical dimension of obesity-induced macromolecular injury. 8-OHdG is the most commonly used marker for ROS-induced genomic and mitochondrial DNA oxidation [260,261]. Elevated placental 8-OHdG levels have been documented in both human and animal studies of maternal obesity, providing evidence of oxidative DNA injury in this tissue [302,303]. Nevertheless, its specificity to obesity-related oxidative stress is only moderate, as DNA oxidation may arise from diverse sources beyond obesity. Moreover, urinary 8-OHdG concentrations are susceptible to confounding influences from multiple physiological and lifestyle factors. Renal function critically affects 8-OHdG excretion efficiency, while physical activity and dietary factors, including alcohol consumption and smoking status, can influence 8-OHdG levels [262,263]. The heterogeneity of detection methodologies, including ELISA, HPLC, and LC-MS/MS, further complicates cross-study comparisons. An 18-laboratory international ring trial demonstrated that ELISA values averaged 4.2-fold higher than those from chromatographic methods, with poor inter-method correlation [304]. Furthermore, the biological variability of 8-OHdG within individuals complicates the interpretation of single time point measurements, a practical limitation frequently overlooked in cross-sectional studies. The coefficient of variation is 17% for 24 h excretion and 20% for creatinine-corrected first-morning samples [304]. The interpretation of urinary 8-OHdG is further complicated by the fact that unchanged excretion at increased oxidative burden does not rule out decreased repair capacity and accumulation of 8-OHdG in DNA, and no significant correlation between urinary 8-OHdG and DNA 8-OHdG has been consistently demonstrated [305]. These confounders necessitate careful adjustment in both experimental design and data interpretation when applying this biomarker to obesity research.

In summary, although F2-isoprostanes and 8-OHdG currently represent the best-validated biomarkers for assessing oxidative stress in obesity, both possess inherent limitations, including the lack of obesity-specific reference ranges, the absence of validated cutoff values for metabolic risk stratification, and insufficient large-scale clinical validation in obese populations.

### 3.2. Supportive but Non-Specific Biomarkers

A broader panel of biochemical markers has been extensively employed in obesity research. However, these markers lack sufficient specificity for obesity-related oxidative stress, are substantially confounded by other pathological conditions, or exhibit limited analytical reliability.

Lipid peroxidation markers, including malondialdehyde (MDA), 4-hydroxynonenal (4-HNE), and thiobarbituric acid reactive substances (TBARS), are major end-products of polyunsaturated fatty acid oxidation [29,256]. Elevated levels of these markers have been consistently observed in obese individuals and correlate with the degree of adiposity [306]. However, MDA measured by the TBARS assay lacks specificity [264], as numerous non-MDA substances react with thiobarbituric acid. 4-HNE requires LC-MS/MS, limiting widespread application [266]. Both markers are influenced by dietary lipid composition and are not specific to obesity, as they are elevated in cardiovascular disease, liver disease, and cancer [265]. The TBARS assay has been criticized for its lack of specificity and reproducibility; different laboratories report widely varying baseline values, and the assay is sensitive to hemolysis and sample storage conditions [307]. For MDA quantification by HPLC, while more specific than TBARS, the procedure is more time-consuming and requires specialized equipment, limiting its widespread adoption. The practical implication is that while MDA and TBARS are easy and inexpensive to measure, their poor analytical validity makes them unsuitable for clinical applications. These markers should be interpreted with caution in any clinical study and are best used as exploratory research tools rather than definitive measures of oxidative stress.

Protein oxidation markers, including protein carbonyls, advanced glycation end products (AGEs), and oxidized low-density lipoprotein (ox-LDL), are stable indicators of irreversible protein oxidative injury [267,268]. Elevated levels in obese individuals correlate with IR and inflammatory cascades [31]. Ox-LDL also exerts critical pathogenic effects in obesity. Specifically, it not only serves as a marker of oxidative stress but also participates in the pathogenesis of atherosclerosis, while AGEs activate the NF-κB pathway via their receptor RAGE to promote inflammatory responses [271,272]. The increased abundance of small, dense LDL particles in obese subjects renders lipoproteins more susceptible to oxidation [308]. The resultant ox-LDL activates inflammatory signaling via the LOX-1 receptor and promotes macrophage foam cell formation, a pivotal event underlying obesity-associated atherosclerosis [309]. However, protein carbonyls measured by the DNPH assay are influenced by age and sample handling [269]. AGEs reflect cumulative metabolic stress from both oxidative and glycemic pathways [270]. Ox-LDL levels are strongly influenced by circulating lipid profiles [273]. The absence of standardized reference materials and internationally recognized calibrators further limits the comparison of results across laboratories and studies. From a clinical perspective, none of these markers can be recommended as standalone oxidative stress biomarkers for individual patient assessment. However, they may provide useful supplementary information when interpreted in the context of multiple markers and clinical presentation.

Markers related to enzymatic and non-enzymatic antioxidant systems also provide supplementary insights into obesity-associated redox dysregulation. Gamma-glutamyl transferase (GGT), a key enzyme involved in glutathione metabolism, serves as an independent predictor of metabolic syndrome, T2DM, and hypertension when abnormally elevated [274]. Uric acid exerts dual biological effects in a context-dependent manner: it functions as an antioxidant under aqueous physiological conditions yet shifts toward a pro-oxidant role within the hydrophobic, ROS-enriched microenvironment of hypertrophic adipocytes in obesity [275]. Elevated serum ferritin, frequently observed in patients with metabolic syndrome without evidence of iron overload, correlates positively with IR and hepatic dysfunction, predominantly reflecting obesity-triggered inflammatory activation rather than simple iron accumulation [276]. Although these markers are readily measurable by routine clinical chemistry assays, GGT lacks specificity due to elevation in hepatobiliary disease. Uric acid and ferritin are confounded by renal function and iron storage disorders, respectively. The biological variability of these markers is also a significant consideration; serum uric acid, for example, exhibits diurnal variation, with peak levels in the early morning and a trough in the late afternoon, and is influenced by dietary purine intake, making single measurements less reliable for assessing oxidative status [310]. These markers are best interpreted as indicators of metabolic and inflammatory status rather than specific oxidative stress biomarkers. Their primary value lies in their widespread availability and low cost, but they should not be used as isolated measures of oxidative stress.

Adipokines link adipocyte dysfunction to oxidative stress. Adiponectin is markedly downregulated in obese individuals and inversely correlated with systemic oxidative stress biomarkers [277,278]. In contrast, obesity-related hyperleptinemia exacerbates oxidative stress by activating NOX and suppressing the activity of antioxidant enzymes such as paraoxonase-1 (PON-1) [311,312]. The decrease in PON-1 is associated with increased levels of plasma and urinary F2-isoprostane, MDA, and hydroperoxides [313]. Accordingly, the leptin/adiponectin ratio has been proposed as a more sensitive indicator of metabolic syndrome risk than either biomarker alone [277]. A similar composite approach has been applied to other hepatokines and adipokines. Fetuin-A is elevated in metabolic syndrome and correlates positively with pro-inflammatory cytokines [314], and the fetuin-A/adiponectin ratio has been suggested as an even more sensitive index in elderly populations [279]. Elevated PAI-1 has also been consistently observed in metabolic syndrome and correlates with BMI, triglycerides, and IR [280]. However, adipokine-based biomarkers are influenced by age, sex, and menopausal status. More importantly, they reflect general adipocyte dysfunction and inflammation, with oxidative stress representing only one contributing pathway. These markers have limited clinical utility as oxidative stress biomarkers because they are not specific to oxidative damage and are influenced by many factors unrelated to redox status. However, they may provide useful context when interpreted alongside direct oxidative damage markers. The cost-effectiveness of adipokine-based biomarkers is generally favorable due to their availability as commercial ELISA kits, but their lack of specificity limits their clinical utility. Their clinical utility is optimized when interpreted alongside direct oxidative damage markers.

### 3.3. Novel Biomarkers

Advancements in analytical technologies have enabled the identification of emerging biomarkers that mirror mitochondrial dysfunction and epigenetic dysregulation, providing deeper mechanistic insights into the interplay between obesity and redox imbalance. However, these markers remain largely in the research phase, lacking large-scale clinical validation, standardized analytical protocols, and established clinical utility.

mtDNA-related parameters have emerged as promising integrative biomarkers. Reduced mtDNA copy number in peripheral blood correlates with BMI, IR, and metabolic syndrome severity [281,282]. Importantly, mtDNA depletion is reversible, as weight loss induced by bariatric surgery or metformin restores mtDNA copy number [315,316]. Beyond copy number alteration, epigenetic modification of mtDNA, particularly methylation of the displacement loop (D-loop) regulatory region, has gained increasing research attention. D-loop hypermethylation is strongly associated with reduced mtDNA copy number and compromised mitochondrial function in obesity and IR [287]. Furthermore, oxidized mtDNA (ox-mtDNA) released from damaged adipocytes activates TLR9-NF-κB signaling in patients with metabolic syndrome, linking mitochondrial dysfunction to systemic inflammation [288]. Despite these promising findings, mtDNA copy number is influenced by age, sex, and hematological parameters [283,284,285]. A large-scale genomic study of 274,832 individuals demonstrated that blood cell composition explains 23% of mtDNA copy number variance and that adjusting for this covariate eliminates previously reported associations with cardiometabolic diseases. At the same time, nuclear genetic variants at numerous loci substantially shape mtDNA abundance and heteroplasmy levels, indicating that these measurements reflect sampling-site cellular composition and individual genomic background rather than mitochondrial status alone [88]. Technical challenges, including NUMT contamination, further compromise cross-platform comparability, as different bioinformatics pipelines yield divergent results [88]. Current methodologies lack standardization and validated clinical assays [285,286], thereby limiting these markers to research tools until rigorous validation is achieved.

MicroRNAs (miRNAs) have emerged as candidate circulating biomarkers due to their stability and tissue-specific expression patterns. These molecules, also termed mitomiRs or obesity-associated miRNAs, can be reliably detected in plasma, exosomes, and ATs. For example, a study of circulating miR-24-3p in children with obesity reported that this miRNA distinguished obese children from healthy controls with an AUC of 0.951, 91% sensitivity, and 90% specificity, and predicted metabolic syndrome in obese children with an AUC of 0.890 [289]. MiR-34a is significantly upregulated in the AT of obese mice; inhibition of miR-34a alleviates adiposity and improves metabolic profiles by upregulating thermogenic genes, including UCP1 and PGC-1α [290]. MiR-27a/b, also elevated under obese conditions, promotes mitochondrial fragmentation and excessive ROS production by targeting prohibitin, thereby impairing normal adipocyte differentiation [291,292]. MiR-155 is highly enriched in brown AT and suppresses thermogenesis; genetic deletion of miR-155 in female mice confers protection against HFD-induced obesity [293,294]. Additionally, Ramzan et al. identified miR-15a-5p and miR-17-5p as predictive biomarkers for metabolic syndrome, as these miRNAs modulate genes involved in insulin signaling and fatty acid metabolism, but the validation cohort for these findings consisted of only 20 women [317]. Beyond these examples, broader miRNA profiling studies have identified additional circulating miRNAs whose expression levels correlate with obesity-related metabolic traits. For instance, miR-142-3p, miR-140-5p, and miR-222 are among those found to be elevated in obese individuals, with their levels positively associated with MAMs and adverse lipid parameters [318]. However, the cross-sectional design prevents the determination of causality, and the study did not evaluate longitudinal predictive value or validate findings in an independent cohort.

Despite these promising findings, the clinical translation of miRNA biomarkers faces several challenges. First, for candidate miRNAs intended for clinical biomarker application, analytical validity remains incompletely established. The study evaluating miR-24-3p as a clinical diagnostic marker did not report coefficients of variation (CVs) or follow standardized MIQE guidelines for qPCR-based biomarker studies [289]. This gap is critical because CVs are essential for assessing assay reproducibility, and the MIQE guidelines provide a framework for ensuring the reliability and comparability of qPCR-based diagnostic tests. Second, the validation cohort for miR-15a-5p and miR-17-5p comprised only 20 women [317]; the miR-24-3p study lacked an independent validation cohort [289]; and the cross-sectional study of 250 adolescents did not validate its findings in a separate cohort [318]. Third, the biological variability of circulating miRNAs, influenced by diet, circadian rhythm, and recent physical activity, has not been systematically characterized, complicating the interpretation of single measurements. Finally, many studies are cross-sectional and cannot distinguish whether miRNA dysregulation is a cause or consequence of metabolic dysfunction. These markers are firmly in the research domain and will require substantial additional validation, including established reference ranges, validated cutoff values for clinical decision-making, and cost-effectiveness data, before clinical implementation can be considered.

High-throughput omics approaches further advance the discovery of obesity-related oxidative stress biomarkers. Redox proteomics and metabolomics allow large-scale profiling of oxidized proteins and lipids. Rather than merely assessing global oxidative burden, these approaches can detect specific oxidative post-translational modifications (Ox-PTMs) on individual proteins, including carbonylation, S-glutathionylation, and tyrosine nitration, thereby constructing a refined landscape of cellular redox perturbation in obesity [297,298]. Similarly, advanced lipidomics enables the profiling of specific OxPLs such as OxPAPC, which serve not only as sensitive biomarkers but also as bioactive mediators that drive macrophage polarization toward a pro-inflammatory phenotype [299,300]. Although these high-throughput methodologies require specialized instrumentation and are not yet applicable to routine clinical practice, they hold great potential for novel biomarker discovery and systematic interpretation of molecular mechanisms underlying obesity-related oxidative stress. The analytical validity of omics-derived markers is limited by batch effects, platform variability, and a lack of standardized data-processing pipelines. These approaches are exclusively research tools at present and are far from clinical implementation.

In summary, the biomarkers discussed above span a spectrum from clinically validated markers (F2-isoprostanes, 8-OHdG) to supportive but non-specific markers (MDA, protein carbonyls, AGEs, ox-LDL, GGT, uric acid, ferritin, and adipokines) and emerging research tools (mtDNA parameters, miRNAs, and omics-derived markers). Despite their individual utility, all currently available biomarkers share common translational challenges, including methodological heterogeneity, confounding factors, and the absence of standardized reference ranges and validated cutoff values for obesity-specific applications. The specific research question and available resources must guide the choice of biomarker for clinical obesity research. Validated markers offer the highest analytical validity but are expensive and technically demanding, making them suitable for research studies requiring high data quality but impractical for routine clinical use. Supportive markers are more accessible and affordable, but their poor specificity limits their utility to supplementary roles in multi-marker panels. Novel markers hold substantial promise but lack the analytical and clinical validation required for application beyond research settings.

For clinical implementation, none of the currently available oxidative stress biomarkers meet all the criteria for routine use: established reference ranges, validated cutoff values for clinical decision-making, evidence of clinical utility in guiding patient management, and cost-effectiveness. The biomarkers closest to clinical implementation are F2-isoprostanes and 8-OHdG, which have been most extensively validated analytically and are most strongly associated with clinical outcomes; however, they remain limited by cost and technical requirements. At present, oxidative stress biomarkers in obesity are best utilized in research settings, where they can provide mechanistic insights and serve as secondary endpoints in clinical trials. Future progress toward clinical translation will require large-scale prospective studies to establish reference ranges, validate cutoff values for clinical decision-making, and demonstrate that biomarker-guided interventions improve patient outcomes. Future research priorities for oxidative stress biomarkers in obesity should focus on: (1) establishing standardized protocols for sample collection and analysis to reduce inter-laboratory variability; (2) determining obesity-specific reference ranges and clinically validated cutoff values; (3) conducting large-scale prospective studies to assess the predictive value of these biomarkers for hard clinical endpoints; (4) evaluating the cost-effectiveness of biomarker-guided patient stratification and therapeutic monitoring; and (5) validating candidate markers in independent cohorts with diverse populations, including different ages, sexes, ethnicities, and metabolic phenotypes. Without progress in these areas, the translation of oxidative stress biomarkers from research discovery to clinical practice will remain limited. A rational multi-marker approach is therefore preferable to reliance on any single biomarker, and future studies should prioritize the establishment of standardized protocols, obesity-specific reference ranges, and large-scale prospective validation to bridge the gap between discovery and clinical translation.

## 4. Therapeutic Strategies Targeting Obesity-Associated Oxidative Stress

### 4.1. Lifestyle Interventions: Antioxidant-Oriented Modifications

Lifestyle intervention remains the most effective and safest approach for improving mitochondrial function and reducing oxidative stress in obesity [319]. Critically, these interventions do not function as direct ROS scavengers. Instead, they target the upstream drivers of oxidative stress, nutrient overload, and mitochondrial dysfunction, thereby reestablishing endogenous redox homeostasis [320,321]. This mechanistic distinction is fundamental: lifestyle interventions correct the original cause of oxidative stress rather than attempting to neutralize its downstream consequences, which may explain why they have consistently demonstrated efficacy where exogenous antioxidant supplementation has failed. Calorie restriction (CR) increases the NAD^+^/NADH ratio to activate sirtuin 1 (SIRT1), while the elevated AMP/ATP ratio activates AMPK. Both SIRT1 and AMPK subsequently activate PGC-1α, leading to mitochondrial biogenesis [322,323]. The effects of CR on oxidative stress have been evaluated across multiple human trials. Six months of 25% CR in overweight adults (*n* = 48, BMI 25–30 kg/m^2^) reduced body weight by 10.4%, decreased fasting insulin and core body temperature, and reduced DNA damage, while inducing a metabolic adaptation whereby 24 h energy expenditure fell by 135 kcal per day, approximately 6% more than predicted from weight loss alone [324]. Skeletal muscle biopsies from the same trial revealed increased mitochondrial DNA content and upregulation of PGC-1α, NRF-1, and TFAM, consistent with the activation of mitochondrial biogenesis pathways [325]. Two years of CR (*n* = 218, BMI 22–28 kg/m^2^) sustained these metabolic benefits and reduced urinary F2-isoprostanes (13–17%), a validated marker of systemic oxidative stress. However, the actual CR achieved was approximately 12%, substantially below the prescribed 25% target, highlighting the challenges of long-term adherence in free-living conditions [326]. These findings have been extended to older populations with obesity. In overweight and obese older adults (65–84 years, mean BMI 33.8 kg/m^2^), a 3-year dietary intervention with mild CR (250 kcal/day) showed that weight loss greater than 10% reduced hs-CRP by 59.4% and hs-IL6 by 33.0%, while increasing adiponectin by 53.7% [327]. In overweight female office workers with low physical activity, a 3-month 30% CR improved HOMA-IR by up to 45%, with greater improvements observed in participants who abstained from snacking [328]. A comprehensive review integrating these data concluded that CR is superior to exercise in attenuating metabolic rate and oxidative stress [329]. Mechanistically, animal studies have implicated SIRT1/GPx4-dependent antioxidant pathways in CR-mediated protection [330]. However, several methodological considerations should inform the interpretation of these findings. First, these trials enrolled healthy, non-obese adults (BMI 22–28 kg/m^2^, mean age 38 years, 70% female), limiting generalizability to older, obese, or metabolically compromised populations. Second, the actual CR achieved at 24 months was approximately 12%, substantially below the prescribed 25% target, suggesting that observed reductions in oxidative stress biomarkers may underestimate the true effect of sustained CR [326]. Third, reliance on surrogate endpoints—urinary F2-isoprostanes [326], DNA damage markers [324], and mitochondrial biogenesis transcripts [325]—provides evidence for reduced oxidative damage but does not directly address whether CR extends human lifespan or reduces cardiovascular mortality [326]. Fourth, discordant responses across markers—DNA damage reduced but protein carbonylation unchanged [324]—suggest that CR may selectively affect certain oxidative damage types. Fifth, the correlation between changes in leptin and F2-isoprostanes [326] suggests that the antioxidant effects of CR are mediated, at least in part, by reductions in adiposity, raising the question of whether similar benefits could be achieved through other weight-loss modalities. Finally, the post hoc analysis in older adults [327] and the female office worker study [328] lacked control groups, limiting causal inference. These considerations underscore the need for caution in extrapolating from biomarker changes to clinical benefits and highlight the importance of long-term follow-up studies with hard clinical endpoints. Exercise training activates AMPK, p38 MAPK, and calcium-dependent signaling pathways, which also converge on PGC-1α activation [331,332]. A critical distinction is that exercise-induced ROS are transient and of low intensity, serving to upregulate endogenous antioxidant enzymes through a phenomenon termed hormesis. By contrast, chronic, high-level ROS production in obesity causes macromolecular damage and sustains inflammation [333,334]. This hormetic effect illustrates the physiological signaling role of ROS: transient oxidative bursts trigger adaptive responses, whereas sustained oxidative stress is pathogenic. Indiscriminate antioxidant supplementation may blunt these beneficial adaptive responses. This difference explains why regular exercise restores mitochondrial respiratory capacity and reduces oxidative stress biomarkers (TBARS, ferritin) and inflammatory markers (CRP, TNF-α, IL-6) and improves metabolic outcomes (insulin sensitivity, adiponectin levels) in clinical trials [335,336]. In obese pregnancy, maternal exercise prevents placental hypoxia and lipid accumulation, reduces placental oxidative stress, and improves fetal growth outcomes [246,337,338]. For severely obese individuals, bariatric surgery induces substantial and sustained weight loss and restores mitochondrial DNA copy number in both AT and peripheral blood cells [339,340]. Notably, bariatric surgery produces the most dramatic metabolic improvements precisely because it addresses the upstream driver—nutrient overload—rather than attempting to modulate downstream oxidative stress. Collectively, these lifestyle-based interventions demonstrate that improving mitochondrial health through behavioral modification is feasible, although long-term adherence remains a practical challenge.

### 4.2. Pharmacological Interventions

Pharmacological strategies targeting mitochondrial ROS fall into three categories. The first comprises metabolic modulators that indirectly reduce mitochondrial ROS by altering substrate flux and improving insulin sensitivity. The second includes direct mitochondria-targeted ROS scavengers, although many conventional antioxidants have yielded ambiguous clinical results. The third category encompasses multitarget agents that concurrently enhance endogenous defenses and disrupt downstream pathological signaling cascades.

Metabolic modulators represent the most clinically established category. Metformin, at therapeutic concentrations, acts as a mild inhibitor of mitochondrial complex I, thereby increasing the AMP/ATP ratio, activating AMPK, and promoting PGC-1α-dependent mitochondrial biogenesis, ultimately reducing mitochondrial ROS production [341,342,343]. In obese and overweight patients, real-world studies have demonstrated that metformin achieves clinically meaningful weight loss, with average reductions of approximately 5.8 kg (5.6%) over 6 months [344]. Preclinical evidence further indicates that metformin inhibits DRP1-mediated mitochondrial fission and attenuates ER stress-associated NLRP3 inflammasome activation, suggesting that its antioxidant effects involve multiple mechanisms [345]. Notably, metformin acts primarily through mild inhibition of complex I, which does not involve direct ROS scavenging but rather reduces the substrate flux that drives excessive ROS production. This indirect approach may explain its superior clinical efficacy compared to direct antioxidants. Thiazolidinediones (pioglitazone, rosiglitazone) activate PPAR-γ, which promotes mitochondrial biogenesis in AT through PGC-1α induction [346,347]. These agents also directly inhibit the mitochondrial pyruvate carrier, reducing TCA cycle flux and thereby decreasing ROS production [348]. Despite their clinical efficacy, adverse effects, including fluid retention and cardiovascular risk, have limited their use, and their impact on oxidative stress biomarkers in humans remains inconsistent. This inconsistency likely reflects the tissue-specific nature of PPAR-γ effects. While beneficial in AT, promoting adipocyte differentiation and insulin sensitization [349], thiazolidinediones may have divergent effects in other tissues such as muscle, liver, vascular endothelium, and immune cells [350]. Studies have shown that PPARγ’s transcriptional network is cell-specific and conditional, regulated by post-translational modifications that modulate its interaction with transcriptional coregulators [351]. This underscores the importance of cell- and tissue-specific redox regulation, a factor rarely considered in the design of clinical trials for thiazolidinediones, in which off-target effects, including fluid retention, weight gain, and potential cardiovascular risks, have been consistently observed. Sodium-glucose cotransporter 2 (SGLT2) inhibitors have demonstrated senolytic effects that directly target the source of oxidative stress in obesity. In HFD-fed mice, canagliflozin treatment for 7 days reduced the VAT senescence burden, as evidenced by decreased SA-β-gal activity, downregulation of p53, p21, and p16, and reduced SASP factors, including TNF-α and CCL2, alongside diminished ROS production and attenuated AT inflammation [352]. However, in human studies, the effects of SGLT2 inhibitors on oxidative stress biomarkers in non-diabetic overweight populations remain limited, as most randomized controlled trials have focused on patients with T2DM. This illustrates the challenge of translating findings from diabetic populations to broader obesity populations and underscores the importance of patient selection based on metabolic phenotype, a key aspect of disease heterogeneity that is frequently overlooked. Metabolic status and BMI are dissociable: approximately one-third of obese individuals are metabolically healthy, whereas nearly one-quarter of normal-weight individuals exhibit metabolically unhealthy profiles [353,354]. These observations indicate that metabolic phenotype, rather than BMI alone, should guide patient selection in clinical trials; however, such stratification is rarely implemented. The concept of disease “endotypes” has been proposed to address this heterogeneity, advocating a paradigm shift from conventional trial designs toward phenotype-based precision approaches [355].

Direct mitochondria-targeted ROS scavengers constitute the second category, designed to overcome the pharmacokinetic limitations of conventional antioxidants. MitoQ consists of ubiquinone conjugated to a lipophilic triphenylphosphonium cation, enabling its selective accumulation in mitochondria [356]. Preclinical studies have shown that MitoQ reduces mitochondrial ROS production and suppresses NLRP3 inflammasome activation [357,358]. Mitochondria-targeted antioxidants such as MitoQ and MitoVitE are more effective at reducing oxidative damage in mitochondria [359,360,361]. Nevertheless, this advantage has not been consistently replicated in intact human tissues due to pharmacokinetic limitations, including intestinal absorption, first-pass hepatic metabolism, and tissue distribution that limit effective intramitochondrial concentrations [362]. Oral bioavailability of MitoQ is approximately 10%, constrained by cellular metabolism and efflux transporters that may result in significant first-pass effects [363]. This bioavailability limitation is a recurring theme across antioxidant interventions. Even compounds designed for mitochondrial targeting fail to achieve adequate concentrations at the site of ROS production. The pharmacokinetic barrier is frequently underestimated in preclinical-to-clinical translation, as rodent studies often administer compounds via routes or doses that are not feasible in humans [364]. Mito-TEMPO is a mitochondria-targeted superoxide dismutase mimetic, and SS-31 (elamipretide) stabilizes ETC supercomplexes by binding to cardiolipin [365,366]. Both agents show profound effects on mitochondrial morphology and ROS in animal models, reducing NLRP3 inflammasome activation and M1 polarization in ox-LDL-stimulated macrophages [367]. However, human data are largely confined to small phase II trials in primary mitochondrial myopathies, with no approved indications for obesity or metabolic syndrome. The limited human data for these agents highlight the substantial gap between preclinical promise and clinical application, reflecting pharmacokinetic challenges and the absence of pharmacodynamic biomarkers to confirm target engagement, a deficiency that severely hampers the interpretation of negative trials. In contrast to these mitochondria-targeted compounds, conventional untargeted antioxidants, including vitamins C and E and N-acetylcysteine, have not consistently demonstrated efficacy in obesity-related metabolic dysfunction [368]. This limitation likely reflects their inability to achieve sufficient concentrations at mitochondrial sites of ROS production and their failure to selectively neutralize pathogenic ROS without disrupting physiological redox signaling. These observations underscore both the conceptual advantage of mitochondrial compartmentalization, which enables targeted compounds to reach mitochondrial concentrations substantially higher than those of non-targeted alternatives, and the fundamental limitation of the ‘antioxidant’ paradigm. The goal is not to eliminate ROS entirely but to restore redox balance, and non-selective scavenging cannot distinguish between physiologically essential and pathologically excessive ROS.

### 4.3. Multitarget Agents

Multitarget agents that concurrently enhance endogenous antioxidant defenses and disrupt downstream pathological signaling cascades, such as the NRF2 activator CDDO-Me, upregulate a battery of NRF2-dependent antioxidant enzymes, including superoxide dismutase, glutathione peroxidase (GPx), heme oxygenase-1 (HO-1), and NAD(P)H: quinone oxidoreductase-1 (NQO1), thereby providing coordinated antioxidant defense [369,370]. Reduced NRF2 activity has been shown to contribute to major aging phenotypes, including mitochondrial dysfunction and cellular senescence [371]. In HFD-induced obese mice, chronic CDDO-Me administration prevented visceral fat accumulation and significantly reduced the expression of oxidative stress-activated kinases, including JNK and ERK, as well as the stress-responsive proteins STAT3 and Akt, in AT [372,373]. These changes were accompanied by the restoration of the redox-sensitive anti-inflammatory regulator IκBα and a decrease in TNF-α levels [373]. Beyond AT, CDDO-Me prevented hypothalamic oxidative stress and consequent inflammation by reducing TNF-α and IL-6 and restored leptin signaling through the JAK2-Akt-FOXO1 pathway, thereby attenuating leptin resistance and reducing energy intake [372]. In the phase 3 BEACON trial (*n* = 2185), CDDO-Me treatment resulted in significant weight loss (5.7 kg) and improved glycemic control in overweight patients with T2DM [374]. However, the trial was terminated early due to increased heart failure events, underscoring the critical need for dose optimization and safety monitoring in clinical translation of NRF2-targeted therapies. This case offers critical insights into the challenges of redox-based therapeutics. The marked discrepancy between robust preclinical efficacy and adverse clinical outcomes likely reflects the narrow therapeutic window of certain antioxidant interventions. Moreover, it underscores the importance of tissue-specific effects: while NRF2 activation may confer benefits in AT and the hypothalamus, it appears detrimental in cardiovascular tissues, highlighting the necessity of tissue-selective targeting strategies. The BEACON trial further demonstrates the perils of relying on surrogate endpoints in large-scale trials without rigorous safety monitoring. Targeting ROS-sensitive signaling pathways represents an alternative approach. MCC950, a direct NLRP3 inhibitor, prevents inflammasome assembly and IL-1β release, improving insulin sensitivity in animal models [375]; however, hepatotoxicity has terminated its clinical development [376,377]. DRP1 inhibitors (Mdivi-1, P110, and dynasore) inhibit mitochondrial fragmentation and reduce ROS production in diabetic models [378,379,380]. In skeletal muscle from HFD-induced obese mice, Mdivi-1 reduced Drp1 translocation to mitochondria, lowered H_2_O_2_ content (elevated by 41.2% in HFD-fed mice), and restored insulin-stimulated Akt phosphorylation and glucose tolerance [381]. These findings were replicated in human myotubes from obese IR individuals, where Mdivi-1 restored insulin-stimulated glucose uptake [381]. Separately, the DRP1 inhibitor Mdivi-1 has been shown to suppress DRP1 phosphorylation at Ser616, thereby reducing excessive mitochondrial fragmentation and mito-ROS accumulation in various disease models [367]. In white AT from ob/ob mice, Mdivi-1 restored mitochondrial dynamics, enhanced complex I activity, and induced browning via UCP-1 upregulation, reducing blood glucose by 50% without affecting body weight [382]. Notably, mitochondrial respiration remained unchanged, indicating that the insulin-sensitizing effects of Drp1 inhibition are mediated through reduced oxidative stress rather than enhanced respiratory capacity, highlighting the therapeutic potential of rebalancing mitochondrial dynamics in obesity. However, the specificity of Mdivi-1 has been questioned, and the long-term safety of DRP1 inhibition in humans is unknown, as DRP1 is essential for mitochondrial quality control in multiple tissues, including the brain and heart. This underscores the challenge of translating mechanistic insights from preclinical models into safe human therapies.

Dietary compounds have attracted attention due to their safety profiles and pleiotropic bioactivities. Resveratrol has demonstrated anti-obesity and insulin-sensitizing effects. In obese men, 30 days of resveratrol supplementation at 150 mg per day improved insulin sensitivity, as indicated by reduced HOMA-IR, lower systolic blood pressure, and reduced intrahepatic lipids, while activating AMPK and increasing SIRT1 and PGC-1α protein levels in skeletal muscle [383]. Emerging evidence suggests that resveratrol’s anti-obesity effects are mediated by gut microbiota-derived 4-hydroxyphenylacetic acid, which activates SIRT1 signaling and induces WAT browning by upregulating UCP1 and PGC-1α in HFD-fed mice [384]. Direct oxidative stress biomarkers were measured in an 18-week animal study, where resveratrol restored manganese superoxide dismutase (MnSOD) expression, a key mitochondrial antioxidant enzyme, in epididymal WAT [385]. Despite these promising preclinical and short-term human data, resveratrol’s poor oral bioavailability and extensive first-pass metabolism have limited its clinical translation, and the optimal dosing and formulation remain unresolved. The marked contrast between robust preclinical efficacy and modest human effects exemplifies the pharmacokinetic barrier that plagues many antioxidant interventions. Lycopene activates the PI3K/Akt/NRF2 pathway and inhibits NOX-mediated ROS production, thereby reducing oxidative stress biomarkers (TBARS, 8-iso-PGF2α) and improving metabolic outcomes (insulin sensitivity, hepatic steatosis) in animal models [386,387,388]. Epidemiological evidence supports an inverse association between serum lycopene levels and oxidative stress biomarkers in metabolic syndrome [389]. Curcumin activates the NRF2/HO-1 antioxidant pathway, promotes ROS scavenging, and inhibits MAPK signaling, thereby alleviating oxidative stress in metabolic tissues [390]. In HFD-fed mice, curcumin administration improved glucose tolerance and insulin sensitivity independent of body weight changes. Mechanistically, curcumin restored impaired NRF2 signaling in skeletal muscle by increasing NRF2 nuclear translocation and upregulating the downstream antioxidant enzyme HO-1, leading to reduced malondialdehyde and ROS levels, particularly in the mitochondrial fraction [250]. In obese patients, however, the translational evidence remains limited. A randomized crossover trial reported that curcuminoid supplementation significantly reduced serum triglycerides, although no changes were observed in direct oxidative stress [391]. Astaxanthin has demonstrated antioxidant and metabolic benefits in obesity. In obese males, 12 weeks of high-intensity functional training combined with astaxanthin supplementation (20 mg/day) significantly reduced body weight and body fat percentage and improved lipid profiles and IR, with the combined intervention showing superior effects to either intervention alone [392]. Mechanistically, astaxanthin is suggested to reduce oxidative stress through increasing total antioxidant capacity and superoxide dismutase activity while inhibiting NF-κB activation to suppress pro-inflammatory cytokines [393]. In HFD-induced obese rats, astaxanthin administration reduced body weight, improved lipid profiles, and attenuated hepatic steatosis, while downregulating AT miRNA-222 and miRNA-378 and suppressing TNF-α and IL-6 [394]. However, in patients with coronary artery disease, 8 weeks of astaxanthin supplementation (12 mg/day) only reduced total cholesterol and LDL-cholesterol, without affecting SIRT1, TNF-α, or glycemic indices [395]. These divergent results highlight the importance of study population, disease context, and the distinction between biomarker changes and clinically meaningful outcomes, underscoring the heterogeneity of patient populations and the need for patient selection. While the antioxidant properties of astaxanthin are well-documented in preclinical studies, clinical evidence for its direct effects on oxidative stress biomarkers in obesity remains limited. Butyrate, a gut microbial metabolite, increases mitochondrial uncoupling and improves energy metabolism efficiency [396,397]. In HFD-induced obese mice, butyrate administration alleviated hepatic steatosis and reduced body weight [398,399]. As a histone deacetylase inhibitor, butyrate enhances H3K9 acetylation on the PPARα promoter to upregulate fatty acid β-oxidation while promoting PPARα-p65 interaction to suppress NF-κB-mediated inflammation [398]. Butyrate also activates NRF2-mediated antioxidant defense, as evidenced by increased NQO1, GST, and GSH/GSSG ratio, while reducing mitochondrial H_2_O_2_ production [398]. In AT, butyrate upregulates PKA-mediated lipolysis and mitochondrial oxidative phosphorylation [399]. At the metabolic level, butyrate promotes mitochondrial uncoupling via UCP2, elevating the AMP-to-ATP ratio and activating AMPK to suppress de novo lipogenesis while driving fatty acid oxidation [400]. Translating these preclinical findings to humans, a randomized controlled trial in children with obesity demonstrated that 6-month oral butyrate supplementation, as an adjunct to standard care, significantly reduced BMI and improved insulin sensitivity and miR-221 levels, a regulator of adipose inflammation [401]. The positive butyrate data in children are promising, but the heterogeneity of the gut microbiome across individuals suggests that the efficacy of butyrate supplementation may vary substantially based on baseline microbiome composition. This variability may contribute to the inconsistent results observed across studies and highlights the need for microbiome-based patient stratification.

Epigenetic modulation via antisense oligonucleotides or miRNA sponges targeting miR-34a, miR-155, and miR-125b-5p has been shown to restore thermogenic gene expression and improve mitochondrial function. For example, inhibition of miR-34a increases UCP1 and PGC-1α expression, elevates mitochondrial DNA copy number, and reduces adiposity in HFD-induced obese mice [290,293,402]. However, delivery of nucleic acid-based therapeutics to adipocytes remains challenging due to the presence of large intracellular lipid droplets [403]. This delivery challenge exemplifies a broader barrier for emerging strategies: the physicochemical properties of the therapeutic must be matched to the target tissue, and AT presents unique obstacles due to its lipid-rich environment. Without addressing these delivery barriers, even the most promising agents may fail to achieve clinical translation. Other emerging strategies include mesenchymal stem cell transplantation, which operates via mitochondrial transfer to damaged cells; however, mesenchymal stem cells derived from obese donors exhibit impaired mitophagy and reduced mitochondrial transfer capacity [404]. This observation has important implications, as autologous stem cell therapies may be compromised in obese patients precisely because the donor cells are derived from a metabolically compromised environment, illustrating how disease heterogeneity directly impacts therapeutic efficacy. Maternal serine supplementation during pregnancy may prevent transgenerational transmission of oxidative damage, but the epigenetic mechanisms require further elucidation [405]. Mitochondrial transplantation remains highly experimental, with major technical challenges in isolation, preservation, and tissue targeting [406]. A common feature of these emerging strategies is the substantial gap between the mechanistic promise demonstrated in preclinical models and their practical application in human obesity. Future progress will likely require not only the development of more potent and specific interventions but also the establishment of biomarkers to identify patient subgroups most likely to benefit from mechanism-directed therapies, as well as the development of tissue-specific delivery systems that can achieve adequate concentrations at the site of ROS production without off-target effects.

Despite robust preclinical efficacy, antioxidant-based therapies have largely failed to translate into clinical benefits for obesity. This disparity stems from several interrelated factors that collectively explain the translational gap. First, the physiological signaling roles of ROS are often overlooked in the design of antioxidant interventions. ROS are not simply toxic byproducts of metabolism; they are essential second messengers in insulin signaling, immune function, and adaptive stress responses. Indiscriminate scavenging of ROS may therefore disrupt physiological processes that are essential for metabolic health. Second, the pharmacokinetic properties of most antioxidant compounds are inadequate for therapeutic application. Poor oral bioavailability, extensive first-pass metabolism, and limited tissue penetration prevent the achievement of effective concentrations at mitochondrial sites of ROS production. Even when compounds reach target tissues, their intracellular and intramitochondrial concentrations are often insufficient to neutralize pathogenic ROS without affecting essential signaling pools. Third, the tissue- and cell-specific nature of redox regulation means that systemic antioxidant approaches are inherently imprecise. A compound that reduces ROS in one tissue may have minimal effect or even paradoxical pro-oxidant effects in another, reflecting differences in baseline redox status, antioxidant enzyme expression, and metabolic activity. Fourth, human obesity is characterized by substantial heterogeneity in etiology, AT distribution, metabolic health status, genetic background, and inflammatory profiles. The same intervention may benefit a subset of patients with high oxidative stress while being ineffective in those with preserved redox homeostasis. Clinical trials that fail to stratify patients by these factors are unlikely to detect efficacy. Fifth, the timing of intervention is critical yet frequently mismatched between preclinical and clinical studies. Prophylactic administration in young, genetically homogeneous rodents is fundamentally different from therapeutic administration in patients with established obesity and its comorbidities. The former tests prevention; the latter tests treatment. Sixth, the design of clinical trials has significant limitations, including small sample sizes, short intervention durations, the use of surrogate endpoints that may not reflect clinically meaningful outcomes, and the absence of pharmacodynamic biomarkers to confirm target engagement at the mitochondrial level. Without confirmation that the intervention has achieved its intended molecular effect, negative trials are difficult to interpret. Seventh, the absence of validated biomarkers to guide patient selection represents a major barrier to clinical translation. The development of biomarkers that identify individuals with elevated oxidative stress or specific redox vulnerabilities and that monitor target engagement and biological response is essential for the success of future mechanism-directed antioxidant therapies. Addressing these challenges will require patient stratification based on oxidative stress biomarkers, tissue-specific delivery strategies to achieve adequate concentrations at mitochondrial sites of ROS production, pharmacodynamic biomarkers to confirm target engagement at the mitochondrial level, and clinical trial designs that incorporate mechanistic endpoints. In the absence of these developments, the likelihood of successful translation from preclinical promise to clinical practice will remain low. Therapeutic strategies targeting oxidative stress in obesity are shown in Table 3.

## 5. Conclusions

Obesity-induced oxidative stress is a complex pathophysiological process driven synergistically by mitochondrial dysfunction, chronic inflammation, ER stress, and impairment of endogenous antioxidant defense systems. These pathological mechanisms do not function independently but form bidirectional interactive loops to establish a self-amplifying vicious cycle, ultimately facilitating the pathogenesis of IR, T2DM, and non-alcoholic fatty liver disease. Accumulating evidence has shifted the current understanding from regarding oxidative stress merely as a secondary phenomenon of metabolic disorders to recognizing it as a core pathological driver. Nevertheless, it remains unclear why individuals exhibit distinct susceptibilities to obesity-triggered oxidative stress.

Mitochondrial dysfunction occupies a central position in this regulatory network. Under obese conditions, nutrient surplus elevates electron leakage at mitochondrial respiratory chain complexes I and III, leading to excessive ROS production. Meanwhile, imbalanced mitochondrial dynamics characterized by DRP1 upregulation and MFN2 downregulation, together with suppressed PGC-1α-mediated mitochondrial biogenesis, further aggravate mitochondrial structural and functional damage. Paradoxically, several studies have demonstrated that mice carrying mitochondrial DNA mutations are resistant to HFD-induced obesity, and exercise improves insulin sensitivity without necessarily increasing mitochondrial content. These contradictory findings imply that the correlation between mitochondrial function and metabolic health is not simply linear. Instead, greater importance should be attached to mitochondrial adaptive reserve and stress responsiveness, rather than merely evaluating its basal functional level.

ER stress further complicates obesity-associated oxidative stress via the UPR and enhanced ER–mitochondrial communication. Obesity markedly enhances MAM formation in the liver, accompanied by elevated expression of MAM-enriched proteins, including IP3R1, IP3R2, PACS-2, and Sig1R, thereby promoting excessive calcium transfer from the ER to the mitochondrial matrix and resulting in mitochondrial calcium overload and oxidative dysfunction. Notably, short-term high-fat feeding and acute ER stress induction also increase MAM formation, indicating that enhanced MAM coupling may serve as an early adaptive response rather than a purely pathological alteration. This raises a critical question regarding the transition threshold from adaptive compensation to pathological deterioration of MAMs, which may provide a novel theoretical basis for developing therapeutic strategies aiming to restore rather than excessively inhibit MAM function.

The endogenous antioxidant defense system is profoundly compromised in obesity, whereas the inconsistent clinical outcomes of antioxidant supplementation constitute a thought-provoking paradox. The NRF2/Keap1 signaling pathway, as the master antioxidant regulatory axis, is markedly suppressed in obesity, leading to reduced activities of SOD, CAT, and GPx, as well as significant glutathione depletion. However, supplementation with conventional exogenous antioxidants such as vitamin C and vitamin E fails to yield consistent clinical benefits. Such limitations reflect multiple underlying facts: ROS act not only as toxic oxidants but also as indispensable signaling molecules; ROS derived from different subcellular compartments exert distinct physiological and pathological roles; obesity-related oxidative stress originates from upstream metabolic disturbances, including nutrient excess and chronic inflammation. Simply neutralizing ROS without targeting upstream drivers cannot achieve sustained metabolic improvement. Accordingly, optimal antioxidant strategies should focus on subcellularly targeted interventions, combined anti-inflammatory modulation, and enhancement of endogenous antioxidant capacity, rather than relying solely on exogenous antioxidant supplementation.

Obesity-induced oxidative stress can be transmitted across generations through placental oxidative stress, epigenetic modification, and persistent inflammatory priming. Male offspring tend to display more severe metabolic phenotypes than female offspring, suggesting a critical role of sex differences in determining oxidative stress susceptibility. Current preventive and therapeutic strategies generally adopt a one-size-fits-all approach while ignoring sex-specific regulatory mechanisms. Future studies are required to systematically elucidate the differential regulation of mitochondrial dynamics, antioxidant defense, and inflammatory pathways by sex hormones to establish sex-stratified intervention strategies.

From a therapeutic perspective, lifestyle interventions targeting the activation of AMPK, SIRT1, and PGC-1α remain the fundamental approach to restoring mitochondrial function and mitigating oxidative stress. Mitochondria-targeted antioxidants, including MitoQ, Mito-TEMPO, and SS-31, as well as pharmacological modulation of IP3R1/PACS-2-mediated MAM crosstalk, have shown considerable potential in preclinical investigations. Nevertheless, a fundamental challenge remains unresolved: how to selectively intervene in pathologically enhanced oxidative stress without disrupting physiological organelle crosstalk and necessary ROS signaling. Systemic inhibition of DRP1 or NLRP3 may induce adverse effects in high-energy-consuming tissues such as the heart and brain, highlighting the necessity of precise tissue-specific targeting in future drug design and delivery systems.

Novel biomarkers, including mitochondrial DNA copy number, D-loop methylation status, circulating oxidized mitochondrial DNA, and specific miRNAs, provide promising tools for early detection and personalized monitoring of obesity-related oxidative stress. The clinical value of these biomarkers lies not only in auxiliary diagnosis but also in identifying the optimal intervenable time window, enabling early preventive intervention prior to irreversible tissue damage.

Future breakthroughs in this field may arise from three major directions. First, well-designed animal models are required to distinguish causality from mere correlation so as to clarify the exact role of mitochondrial dysfunction in metabolic pathogenesis. Second, the limited efficacy of single-antioxidant or single-target anti-inflammatory therapy indicates that multi-combined strategies integrating metabolic modulation, anti-inflammation, and mitochondrial protection are urgently needed. Third, emerging evidence from maternal obesity intervention implies that the prenatal stage may represent a crucial window for preventing transgenerational metabolic disorders. Further human studies are warranted to determine the optimal developmental stage and duration of intervention. Addressing these issues will facilitate the translational application of basic research into clinical strategies for obesity and oxidative stress-related metabolic diseases.

## Figures and Tables

**Table 1:** Summary of major molecular mechanisms of obesity-associated oxidative stress.

Mechanism Category	Key Pathways	Primary Evidence Level	Key Findings	Unresolved Questions/Critical Considerations	Ref
Mitochondrial dysfunction	ETC leakage, Δψ_m_ collapse, mtDNA damage, PGC-1α suppression	Human observational (cross-sectional); Animal models; Cell culture	↓ ETC activity (Complexes I, III, IV), ↓ cristae density, ↓ mtDNA copy number, ↑ ROS production in skeletal muscle, liver, and AT	Causality unproven in humans (cross-sectional); supraphysiological FFA in cell studies; tissue-specific heterogeneity poorly understood	[52,53,54,55,58,59,60,63,64]
Imbalanced mitochondrial dynamics	↑ DRP1-mediated fission, ↓ MFN2-mediated fusion	Animal models; Human observational	Excessive fission → ETC complex III ↓ >30%, SOD activity ↓, ↑ ROS, mtDNA damage; MFN2 correlates positively with insulin sensitivity, inversely with BMI	Causality unestablished; tissue specificity unknown; Mdivi-1 has off-target effects; both excess fission and fusion can be detrimental.	[68,69,70,73,254]
Antioxidant defense failure	↓ SOD2/GPx/CAT expression, GSH depletion	Human observational; Animal models; Cell culture	↓ SOD/CAT/GPx activity, ↑ GSSG/GSH ratio, ↑ MDA/4-HNE/protein carbonyls/8-OHdG in plasma and tissues	mtDNA damage assays lack standardization; no prospective human data; mtDNA copy number confounded by age/sex/hematology; MDA assays prone to artifacts	[192,193,198,199,200,201,202,203,204,205]
NADPH oxidase activation (NOX4)	NOX4/NF-κB/MCP-1 axis, NOX2 upregulation	Animal models; Cell culture	NOX4 relocalization → NF-κB activation → MCP-1 → macrophage recruitment; global vs. adipocyte-specific NOX4 deletion yields opposing effects on insulin sensitivity	No human adipose NOX data; DPI/apocynin lacks specificity; 3T3-L1 data may not generalize to primary adipocytes	[90,113,114]
Chronic low-grade inflammation	M1 macrophage polarization, TNF-α/IL-6/IL-1β release	Human observational; Animal models	↑ M1/M2 ratio, ↑ pro-inflammatory cytokines, ↑ CRP, TLR4 activation; serum cytokine levels correlate with BMI and visceral fat area	AT macrophage proportion varies widely (15–50%); temporal relationship with IR unresolved; cytokine-BMI correlations modest (r ≈ 0.3–0.4)	[100,101,102,103,104,105,106,110]
ER stress	IRE1/PERK/ATF6 activation, CHOP, JNK	Human observational; Animal models; Cell culture	↑ p-PERK, ↑ p-IRE1, ↑ CHOP, ↑ pro-inflammatory cytokines; hypothalamic ER stress induces leptin and IR	CHOP isoform complexity ignored; chemical chaperones lack specificity; rodent doses exceed feasible human doses	[140,141,142,179,180,181,182]
ER-mitochondrial crosstalk	MAM formation, IP3R-mediated Ca^2+^ transfer	Animal models; Cell culture	↑ ER-mitochondria contact, ↑ mitochondrial Ca^2+^, mPTP opening, apoptosis; knockdown of IP3R1 or PACS-2 normalizes calcium flux and improves glucose tolerance	No human MAM imaging or functional data; comparable MAM expansion in human AT/muscle/β-cells unknown	[141,157,158,159,160,161,162,163]
Inflammation-oxidative stress cycle	NF-κB/NLRP3, NRF2/NF-κB antagonism	Animal models; Cell culture	NF-κB suppresses NRF2 via CBP competition, HDAC3 recruitment, and Keap1 interaction; NRF2 inhibits NF-κB via HO-1; bidirectional amplification loop	NLRP3 regulation by NRF2 context-dependent; most studies use acute ROS bursts, not chronic obesity-like ROS	[122,123,124,125,126,127,128]
Ferroptosis-related antioxidant failure	NRF2-dependent SLC7A11/GPx4, iron homeostasis	Preclinical models; Cell culture	↓ GPx4, ↑ lipid peroxidation, ↑ iron accumulation in metabolic tissues; NRF2 transcriptionally activates SLC7A11, FTH1/FTL, FPN1, and FSP1	Ferroptosis markers not specific; labile iron is hard to measure; ferroptosis has not been demonstrated in human obesity; MDA/4-HNE not specific	[227,228,229,230,231,232,233,234,235,236,237,238,239,240,241,242,243]
Hypothalamic ER stress	PERK/IRE1 activation, leptin/IR	Animal models	↑ p-PERK, ↑ p-IRE1, ↓ leptin sensitivity, ↑ microglial activation; chemical chaperones reduce body weight and improve leptin sensitivity	UPR markers not examined in human hypothalamus; chaperones lack hypothalamic specificity; CSF biomarkers not established	[179,180,181,182]

↓ Decrease; ↑ increase; 4-HNE, 4-hydroxynonenal; 8-OHdG, 8-hydroxydeoxyguanosine; AT, adipose tissue; BMI, body mass index; CAT, catalase; CHOP, C/EBP homologous protein; CRP, C-reactive protein; ER, endoplasmic reticulum; ETC, electron transport chain; FPN1, ferroportin 1; FSP1, ferroptosis suppressor protein 1; FTH1/FTL, ferritin heavy and light chains; GPx, glutathione peroxidase; GSH, reduced glutathione; GSSG, oxidized glutathione; HO-1, heme oxygenase 1; IL, interleukin; IP3R, inositol 1,4,5-trisphosphate receptor; IRE1, inositol-requiring enzyme 1; JNK, c-Jun N-terminal kinase; MAM, mitochondria-associated ER membrane; MCP-1, monocyte chemoattractant protein 1; MDA, malondialdehyde; MFN2, mitofusin 2; mtDNA, mitochondrial DNA; NF-κB, nuclear factor kappa B; NOX, NADPH oxidase; NRF2, nuclear factor erythroid 2-related factor 2; PACS-2, phosphofurin acidic cluster sorting protein 2; PERK, PKR-like ER kinase; PGC-1α, peroxisome proliferator-activated receptor gamma coactivator 1-α; ROS, reactive oxygen species; SOD, superoxide dismutase; TLR4, Toll-like receptor 4; TNF-α, tumor necrosis factor alpha; UPR, unfolded protein response.

**Table 2:** Summary of oxidative stress biomarkers in obesity.

Category	Biomarker	Sample Source	Biological Significance	Advantages	Limitations
Clinically Validated	F2-isoprostanes (e.g., 8-epi-PGF2α)	Plasma, urine, tissue	Lipid peroxidation; gold standard	High specificity and stability [255,256]	Requires GC-MS/LC-MS/MS; high cost; confounded by age, sex, ethnicity, smoking [257,258,259]
Clinically Validated	8-OHdG	Urine, serum, tissue	DNA oxidation	Most widely studied DNA oxidation marker [260,261]	Moderate specificity; confounded by renal function, physical activity, diet, smoking; method heterogeneity (ELISA/HPLC/LC-MS/MS) [262,263]
Supportive	MDA/TBARS	Plasma, serum	Lipid peroxidation	Simple, low-cost assay [29]	Poor specificity; confounded by diet; elevated in multiple conditions [264,265]
Supportive	4-HNE	Plasma, tissue	Lipid peroxidation	More specific than MDA	Requires LC-MS/MS; limited accessibility [266]
Supportive	Protein carbonyls	Plasma, serum	Protein oxidation	Reflects widespread protein damage [267,268]	Non-specific; influenced by age and sample handling [269]
Supportive	AGEs	Plasma, serum	Protein oxidation/glycation	Reflects cumulative metabolic stress [270]	Non-specific; reflects both oxidative and glycemic pathways
Supportive	ox-LDL	Plasma, serum	Lipoprotein oxidation; atherosclerosis	Links to cardiovascular pathology [271,272]	Confounded by lipid profiles [273], variable ELISA results
Supportive	GGT	Serum	Glutathione metabolism	Routine clinical assay; low cost [274]	Non-specific (hepatobiliary disease)
Supportive	Uric acid	Serum	Dual antioxidant/pro-oxidant	Routine clinical assay [275]	Dual role complicates interpretation; confounded by renal function
Supportive	Ferritin	Serum	Iron metabolism; inflammation	Routine clinical assay [276]	Reflects inflammation more than oxidative stress
Supportive	Adiponectin	Plasma, serum	Adipocyte function	Mechanistically linked to adipose dysfunction [277,278]	Non-specific; influenced by age and sex
Supportive	Leptin/adiponectin ratio	Plasma, serum	Composite adipokine dysfunction	May improve predictive value [277]	Requires two measurements; additive cost
Supportive	Fetuin-A/adiponectin ratio	Plasma, serum	Hepatokine-adipokine composite	Sensitive in elderly populations [279]	Limited cross-validation
Supportive	PAI-1	Plasma, serum	Fibrinolysis regulation	Correlates with metabolic syndrome components [280]	Non-specific to oxidative stress
Emerging	mtDNA copy number	Peripheral blood	Mitochondrial biogenesis	Mechanistically relevant [281,282]	Confounded by age, sex, blood counts; no standardization [283,284,285,286]
Emerging	D-loop methylation	Peripheral blood, tissue	Mitochondrial epigenetics	Links to obesity and IR [287]	Requires bisulfite sequencing; research-only
Emerging	Oxidized mtDNA	Plasma, serum	mtDNA damage; DAMP signaling	Links mitochondrial damage to inflammation [288]	Evolving methodology; no validated assays [285,286]
Emerging	MicroRNAs (miR-24-3p, miR-34a, miR-27a/b, miR-155, miR-15a-5p, miR-17-5p, miR-142-3p, miR-140-5p, miR-222)	Plasma, serum, exosomes	Post-transcriptional regulation	Stable; non-invasive [289,290,291,292,293,294,295,296]	Small studies; no standardization; causality unclear
Emerging	Ox-PTMs (carbonylation, S-glutathionylation, nitration)	Tissue, plasma	Protein-level oxidative modifications	Mechanistic insights [297,298]	Requires specialized MS; research-only
Emerging	OxPLs (e.g., OxPAPC)	Plasma, tissue	Bioactive OxPLs	Mechanistic and biomarker value [299,300]	Requires advanced lipidomics; research-only

**Table 3:** Therapeutic strategies targeting oxidative stress in obesity.

Specific Intervention	Population/Model	Intervention Protocol	Study Type	Evidence Strength	Oxidative Stress Biomarkers	Metabolic Outcomes	Ref
Caloric restriction with no snacking	Female office workers with overweight/obesity (*n* = 48, age 20–38); low physical activity during COVID-19 lockdown	3-month CR (30% reduction from baseline); increased PUFA and fiber (>30 g/day); reduced SFA and simple carbohydrates; 4–5 meals/day; no snacking; weekly dietitian supervision	Clinical (quasi-experimental, no control)	Limited (large RCT; direct F2-isoprostane measurement; dose–response; sustained 24-month effects)	Not directly measured (indirect: improved lipid ratios and anthropometrics reflecting reduced inflammation)	↓ Body weight (−8%), ↓ BMI (−7%), ↓ WC (−5.8%), ↓ HC (−3.8%), ↓ WHR (−5.8%), ↓ BFP; ↓ insulin (NS: −6%; S: −37%), ↓ HOMA-IR (NS: −25%; S: −45%), ↓ TC, ↓ LDL-C (NS: −17%; S: −27%), ↓ TG, ↑ HDL-C; improved AIP and lipid ratios	[328]
Caloric restriction (preclinical)	Adult male Sprague-Dawley rats (*n* = 7/group; weight 230 ± 15 g)	4-week CR (60% of ad libitum, i.e., 40% reduction) before contrast medium injection (iopromide, 1.8 g/kg i.v.); SRT1720 (500 mg/kg) and EX527 (500 mg/kg) for mechanistic validation	Preclinical (animal)	Moderate (RCT design; direct DNA damage measurement; small sample size)	↓ ROS (DHE fluorescence), ↓ MDA, ↑ SOD, ↑ GSH/GSSG ratio, ↑ GPx4 expression; effects blocked by SIRT1 inhibitor EX527	Renal function: ↓ serum Cr, ↓ BUN, ↑ CCr; Histopathology: ↓ tubular necrosis, ↓ cast formation; Apoptosis: ↓ TUNEL-positive cells, ↓ cleaved caspase-3; Inflammation: ↓ MPO, ↓ IL-1β, ↓ TNF-α	[330]
Caloric restriction (CALERIE 1)	Healthy overweight adults (*n* = 48, 25 ≤ BMI < 30, age 26–48); sedentary	6-month CR (25% reduction) ± exercise (CREX: 12.5% CR + 12.5% exercise) or LCD (very low calorie to 15% weight loss then maintenance)	Clinical (RCT)	Limited (no control group; small sample; no direct oxidative stress biomarkers)	↓ DNA damage (comet assay, *p* ≤ 0.002);	↓ Body weight; ↓ fasting insulin;	[324]
Caloric restriction (CALERIE 2)	Healthy non-obese adults (*n* = 218; CR = 143, AL = 75; age ~38 years; BMI ~25 kg/m^2^); 70% female	2-year CR (targeted 25% reduction; actual ~10–15%); self-selected diets; no exercise requirement	Clinical (RCT)	Moderate (direct ROS/MDA/GSH measurement; SIRT1 pathway validation; no human data)	Primary: ↓ 2,3-dinor-iPF(2x)-III (12mo: −17%; 24mo: −13%; *p* < 0.01); Secondary: ↓ iPF(2x)-III (12mo: −20%; 24mo: −27%; *p* < 0.05); ↓ isoprostane factor (12mo: −0.358 SD; 24mo: −0.311 SD; *p* < 0.01); changes correlated with ↓ leptin (r_s = 0.326) and ↑ insulin sensitivity (r_s = −0.234)	↓ Body weight; ↓ leptin; ↑ insulin sensitivity; ↓ HOMA-IR; ↓ TC, ↓ TG; ↓ blood pressure; III-series isomers are more sensitive to CR than VI-series	[326]
Aerobic + resistance exercise training	Obese middle-aged men (*n* = 212; E_T = 108, D_T = 104); sedentary; BMI > 25 kg/m^2^; age 35–65 years	12-week supervised exercise training (90 min/session, 3 sessions/week: aerobic fast walking/jogging + resistance training) without dietary restriction; compared with dietary restriction alone	Clinical (retrospective analysis, non-randomized)	Limited (non-randomized; retrospective; biomarkers not primary endpoint)	↓ TBARS; ↓ ferritin	↓ Weight, ↓ WC, ↓ VAT; ↓ TG, ↑ HDL-C; ↓ hs-CRP, ↓ TNF-α, ↓ IL-6, ↓ leptin; ↑ adiponectin; ↓ ALT, ↓ γ-GT; ↓ HOMA-IR;	[336]
Physical activity (observational)	Obese pregnant women (*n* = 96; pre-pregnancy BMI ≥29 kg/m^2^, age 33.3 ± 5.3 years)	Objectively measured MVPA and ST (accelerometry) at <20, 24–28, and 35–37 weeks’ gestation; longitudinal analysis.	Clinical (prospective observational)	Moderate (objective exposure measurement; longitudinal; sex-specific findings)	↓ HSP70 mRNA with higher mean MVPA; ↓ GCLM mRNA with higher mean MVPA; Sex-specific: ↑ HO-1 mRNA with higher ST only in male placenta	MVPA decreased from early to late pregnancy;	[246]
Treadmill exercise (preclinical)	C57BL/6J female mice; HFD-induced obesity (60% fat, 8 weeks pre-mating); pregnant mice (*n* = 6/group)	Daily treadmill exercise during gestation (E1.5-E16.5); intensity: 40–65% VO_2_max; 60 min/session; sedentary controls	Preclinical (animal)	Preliminary (no direct oxidative stress biomarkers; no human data)	Not directly measured (oxidative stress inferred from inflammation and vascular markers); HFD-induced placental hypoxia and inflammation; exercise reversed these effects	Maternal: ↓ weight gain, ↓ glucose, ↓ insulin, ↓ HOMA-IR, ↓ AT, ↑ energy expenditure, ↑ muscle strength; Fetal: prevented fetal overgrowth; Placental: ↑ vascular density (CD34, eNOS), ↑ VEGF, ↑ VEGFR1, ↑ HIF1α, ↑ APLN, ↑ CD31; Inflammation: ↓ TNF-α, ↓ IL-1β; Signaling: ↑ p-AMPK, ↑ p-Akt, ↑ p-IRS-1, ↑ p-Erk, ↑ p-mTOR	[337]
Bariatric surgery	Patients with morbid obesity (*n* = 18, BMI 40.9 ± 6.0) and obesity with T2DM (*n* = 14, BMI 40.3 ± 9.0); healthy volunteers (*n* = 22); 6-month follow-up	Roux-en-Y gastric bypass or sleeve gastrectomy; individualized procedure; 6-month post-surgery follow-up	Clinical (prospective cohort)	Moderate (established clinical procedure; direct mtDNA measurement; small sample)	↓ Urinary mtND-1 and mtCOX-3 (both obesity groups; *p* = 0.006 and *p* = 0.030); ↓ Serum mtCOX-3 (T2DM group only; *p* = 0.039); changes in urinary mtDNA correlated with changes in eGFR;	↓ BMI; ↓ HbA1c; ↓ fasting glucose, ↓ triglycerides, ↓ LDL-C, ↑ HDL-C;	[339]
Metformin	Non-diabetic overweight/obese outpatients (*n* = 154, mean age 37.8 years, mean BMI 35.4 kg/m^2^); 45 untreated controls	Metformin 6 months; dose titration by BMI: BMI < 30: 1500 mg/day; BMI 30–35: 2000 mg/day; BMI ≥ 35: 2500 mg/day; no concomitant diet program; those planning lifestyle changes excluded	Clinical (prospective, non-randomized, controlled)	Limited (non-randomized; no oxidative stress biomarkers)	Not directly measured	↓ BMI; ↓IR	[344]
MitoQ	DSS-induced colitis mice (Balb/c, *n* = 5/group); THP-1 human macrophages; IBD patient PBMCs (*n* = 14)	Animal: MitoQ 500 µM oral, daily, 14 days; Cell: MitoQ 50–150 nM + H_2_O_2_ 5 mM + ATP	Preclinical (animal + cell)	Preliminary (direct ROS and senescence biomarker measurement; no human obesity data)	↓ mtROS (MitoSOX), ↓ MDA (lipid peroxidation), ↓ nitrotyrosine (peroxynitrite-mediated nitration); blocks TXNIP-NLRP3 binding	Animal: Body weight recovered, colon length shortening ↓, bloody stool score ↓, histological inflammation score ↓ (*p* < 0.001), IL-1β and IL-18 ↓; Cell: IL-1β and IL-18 release ↓ dose-dependently	[357]
Canagliflozin (SGLT2 inhibitor)	HFD-induced obese mice (C57BL/6, male, 12–14 weeks old)	Canagliflozin 0.03% *w*/*w* mixed in HFD for 7 days or 4 weeks	Preclinical (animal)	Preliminary (direct mtROS/MDA measurement; mechanistic; no obesity-specific human data)	↓ DHE-positive area (ROS) in WAT; ↓ crown-like structures; ↓ SA-β-gal activity; ↓ p53, ↓ p21/p16 mRNA; ↓ SASP factors (Tnf, Ccl2); ↑ p-AMPK/AMPK; ↓ PD-L1 on senescent cells	↓ Fasting blood glucose; ↓ GTT AUC; ↓ ITT AUC; ↔ Body weight, ↔ gWAT weight (7-day);	[352]
Bardoxolone methyl (CDDO-Me)	Overweight/obese patients with T2DM and stage 4 CKD (*n* = 2185); mean BMI 33.7–33.9 kg/m^2^; 93% had BMI ≥25 kg/m^2^	20 mg once daily oral; randomized, double-blind, placebo-controlled (BEACON phase 3); up to 48 weeks	Clinical (RCT, phase 3; post hoc analysis)	Not Established (phase 3 RCT terminated for safety; no oxidative stress biomarkers measured)	NRF2 activation and NF-κB inhibition are cited as mechanisms (oxidative stress biomarkers not directly measured in this analysis)	↓ Body weight; ↓WC; ↓ HbA1c	[374]
CDDO-Me (preclinical)	C57BL/6J mice; HFD 21 wk (prevention)	CDDO-Me 10 mg/kg/day in drinking water	Preclinical (animal)	Preliminary (anti-inflammatory mechanism via M1→M2 shift; no direct oxidative stress biomarkers; no human data)	Hypothalamus: ↓ TNF-α, ↓ IL-6, ↓ pJNK; ↑ pJAK2, ↑ pAkt, ↑ pFOXO1; ↓ PTP1B, ↓ pAMPK; ↑ BDNF; Plasma: ↓ Leptin	↓ Body weight; ↓ energy intake;	[372]
CDDO-Me (preclinical)	C57BL/6J mice; HFD 21 wk (prevention)	CDDO-Me 10 mg/kg/day in drinking water	Preclinical (animal)	Preliminary (hypothalamic pathway validation; no direct oxidative stress biomarkers; no human obesity data)	Visceral fat: ↓ STAT3, ↓ Akt, ↓ ERK, ↓ JNK; ↑ IκBα; ↓ TNF-α; ↓ F4/80 macrophages	↓ Adipocyte surface area; restored adipocyte number/size distribution; ↑ β3-AR, ↑ pTH/TH, ↑ UCP2	[373]
N-acetylcysteine (NAC)	C57BL/6J mice; HFD 8 weeks; isolated FDB muscle fibers	NAC 10 mM, 1 h ex vivo	Preclinical (ex vivo)	Not Established (ex vivo only; no in vivo translation)	↓ PRDX2 dimerization; ↓ ROS-dependent TXNIP/NLRP3 proximity (PLA); ↓ MDA	Restored insulin-dependent Akt phosphorylation and 2-NBDG uptake in HFD fibers;	[375]
Mdivi-1	ob/ob mice (leptin-deficient obesity + diabetes) vs. wt C57BL/6; epididymal WAT	Mdivi-1 50 mg/kg/d, i.p., 3 days	Preclinical (animal)	Preliminary (mechanistically informative; no direct biomarker measurement; no human data)	Not directly measured;	↓ Blood glucose; ↑ Mitochondrial biogenesis (PGC-1α mRNA, mtDNA, AMPK-P); ↑ Complex I activity; ↑ Glucose and fatty acid oxidation; ↑ UCP-1, ↑ PPARγ; ↓ Adipocyte area	[381]
Mdivi-1	C57BL/6J mice; HFD (45% fat) 5 weeks; gastrocnemius muscle; human myotubes from obese IR individuals	Mdivi-1 20 mg/kg, i.p., every other day, last week of HFD; human myotubes: 20 µM, 12 h	Preclinical (animal + human primary cells)	Preliminary (direct H_2_O_2_ measurement; human myotube validation; no clinical trials)	Skeletal muscle H_2_O_2_: HFD ↑41.2% vs. LFD; Mdivi-1 reversed to LFD levels (*p* < 0.05); Myotube ROS: Obese ↑ vs. Lean; Mdivi-1 ↓	↓ Body weight; ↓ GTT AUC; ↓ ITT blood glucose; ↑ Insulin-stimulated Akt phosphorylation; ↓ Drp1; ↑ Mitochondrial network morphology; ↑ Insulin-stimulated glucose uptake (human myotubes)	[382]
Resveratrol	Healthy obese males (*n* = 11, BMI 31.6 ± 0.7, age 52.5 ± 2.1); randomized crossover	Resveratrol 150 mg/day vs. placebo; 30 days per period; 4-week washout	Clinical (RCT, crossover)	Limited (very small sample; no direct oxidative stress biomarkers)	Not directly measured (no ROS, MDA, SOD, GSH, or F2-isoprostanes); Inflammatory markers: ↓ TNF-α (*p* = 0.04), ↓ leukocytes (*p* = 0.03)	↓ Sleeping metabolic rate; ↓ Systolic BP; ↓ Fasting glucose, ↓ F ↓ HOMA-IR; ↓ ALAT, ↓ Intrahepatic lipid; ↑ AMPK, ↑ SIRT1, ↑ PGC-1α; ↑ Mitochondrial respiration on fatty acid substrates	[383]
Resveratrol (preclinical)	C57BL/6J mice; HFD-induced obesity (60% fat, 16 weeks); antibiotic-treated mice; FMT recipients	Resveratrol 300 mg/kg/day ± antibiotics; 4-HPA 30 mg/kg/day ± EX527 (SIRT1 inhibitor); 16 weeks	Preclinical (animal)	Preliminary (gut microbiota mechanism; SIRT1 pathway validation; no direct oxidative stress biomarkers; no human data)	Not measured (no ROS, MDA, SOD, GSH reported)	Resveratrol: ↓ Body weight, ↓ WAT, ↓ adipocyte size, ↓ Fasting glucose/insulin, ↓ HOMA-IR, ↓ IL-1β/TNF-α/IL-6/LPS, ↑ IL-10; Gut microbiota: ↑ Akkermansia, ↑ Bacteroides, ↑ Blautia, ↓ Lactobacillus; 4-HPA: reversed obesity and glucose intolerance via SIRT1 signaling; effects abolished by EX527	[384]
Resveratrol (preclinical)	Male C57BL/6J mice; high-calorie high-cholesterol diet (HCD)-induced obesity; *n* = 8/group	Resveratrol 400 mg/kg/day, intragastric, 18 weeks	Preclinical (animal)	Preliminary (direct MnSOD restoration; no human data)	MnSOD/Sod2 (mRNA and protein): HCD ↓, resveratrol significantly restored; SIRT1: HCD ↓, resveratrol increased	↓ Body weight, ↓ Epididymal WAT weight, ↑ IκB-α, ↓ Tnfα, ↓ Il1b; ↑ Serum testosterone	[385]
Lycopene	Male C57BL/6J mice; HFD (45% fat); 8 weeks; *n* = 8/group	Lycopene 0.012% in diet (~12 mg/kg/day); 8 weeks	Preclinical (animal)	Preliminary (direct oxidative biomarker measurement; consistent effects; no human data)	↓ TBARS in eWAT; ↓ NOX subunits (Nox4, gp91phox, p22phox, p67phox, p47phox); ↑ Sod1, Gpx1, Cat (*p* < 0.05)	↓ Fasting insulin, ↓ HOMA-IR, improved GTT/ITT; ↓ Plasma TG, ↓ TC; ↓ Adipocyte hypertrophy; ↓ M1/M2 ratio; ↓ TNF, ↓ IL-6, ↓ IL-1β, ↓ CCL2; ↓ Hepatic steatosis, ↓ Hepatic TG/TC/NEFA/TBARS	[388]
Lycopene	Male C57BL/6J mice; HFD (45% fat); 12 weeks; *n* = 10/group	Lycopene 10 mg/kg food/day OR Tomato Powder (equivalent lycopene); 12 weeks	Preclinical (animal)	Preliminary (direct 8-iso-PGF2α measurement; consistent effects; no human data)	↓ 8-iso-PGF2α (plasma): lycopene *p* < 0.01, TP *p* < 0.001; ↓ NF-κB p65/IκB phosphorylation (*p* < 0.05)	↓ TG (*p* < 0.01), ↓ NEFA (lycopene, *p* < 0.01); ↓ Glycemia, ↓ HOMA-IR; ↓ Hepatic steatosis; ↓ Il-6/Tnfα/Ccl2/Ccl5, ↑ IL-10/TGF-β	[388]
Curcumin	Male C57BL/6J mice; HFD (60% fat) 16 wk; *n* = 10/group	Curcumin 50 mg/kg/day, oral gavage, 15 days	Preclinical (animal)	Preliminary (direct mitochondrial MDA and ROS measurement; NRF2/HO-1 validation; no human data)	Serum MDA: HFD ↑, curcumin ↓; Skeletal muscle MDA: HFD ↑ ~2-fold, curcumin reversed (*p* < 0.001); Muscle mitochondrial MDA: HFD ↑ 4-fold, curcumin ↓ (33.07 → 10.05 nmol/mg, *p* < 0.001); ↓ Muscle ROS; ↑ NRF2 nuclear translocation; ↑ HO-1	↓ IPGTT glucose, ↓ Fasting glucose, ↓ Fasting insulin, ↓ HOMA-IR; ↔ Body weight; ↓ Epididymal fat	[250]
Astaxanthin	Obese males (*n* = 60, BMI > 30, age 27.6 ± 8.4); randomized: CG, SG, TG, TSG	Astaxanthin 20 mg/day or placebo; HIFT (CrossFit, 60 min/session, 3 sessions/week); 12 weeks	Clinical (RCT)	Limited (no direct oxidative stress biomarkers measured; biomarkers inferred from literature)	Discussion only (no direct oxidative stress biomarkers measured); authors cite “increases plasma TAC and SOD” from literature; ↓ CTRP9, ↓ CTRP2, ↓ GDF8, ↓ GDF15	↓ Weight, ↓ BMI, ↓ Body fat%, ↑ FFM, ↑ VO2peak; ↓ TC, ↓ TG, ↓ LDL-C, ↑ HDL-C; ↓ Fasting glucose, ↓ Insulin, ↓ HOMA-IR;	[392]
Astaxanthin	CAD patients (*n* = 50, age 58.2 ± 5.5, BMI 30.1 ± 3.2); randomized double-blind placebo-controlled	Astaxanthin 12 mg/day or placebo; 8 weeks; both groups received a low-calorie diet	Clinical (RCT)	Limited (no direct oxidative stress biomarkers; small sample)	↔ TNF-α; ↔ SIRT1;	↓ TC, ↓ LDL-C; ↔ TG, ↔ HDL-C; ↔ FBS, ↔ Insulin, ↔ HOMA-IR; ↓ BMI, ↓ Weight	[395]
Astaxanthin (preclinical)	Male albino rats; HFD-induced obesity (8 weeks); *n* = 10/group	Astaxanthin 50 mg/kg/day oral, 8 weeks	Preclinical (animal)	Preliminary (no oxidative stress biomarkers directly measured; no human data)	↓ TNF-α (liver gene expression); ↓ IL-6 (plasma); ↓ Calprotectin (plasma); ↑ Adiponectin (plasma); ↓ Leptin (plasma); ↓ miRNA-222, ↓ miRNA-378 (AT)	↓ Body weight; ↓ Glucose, ↓ Insulin; ↓ TC, ↓ TG, ↓ LDL, ↑ HDL; reversed fatty liver lesions	[394]
Sodium butyrate	SD rats; HFD-induced NAFLD (9 weeks); *n* = 8/group	Sodium butyrate 300 mg/kg, gavage every other day, 7 weeks	Preclinical (animal)	Preliminary (no oxidative stress biomarkers directly measured; no human data)	↓ IL-1β, ↓ IL-6, ↓ TNF-α (*p* < 0.05); ↓ F4/80 (M1), ↑ CD206 (M2); ↓ p-p65, ↓ p-IKKα/β; ↓ NLRP3, ↓ Cleaved caspase-1; ↓ HDAC1; ↑ H3K9Ac on PPARα promoter; ↑ PPARα-p-p65 interaction	↓ Body weight; ↓ Hepatic TG, ↓ Liver weight; ↑ PPARα, ↑ CPT1, ↑ COX1/COX4, ↑ Complex III/V activity, ↑ ATP; ↓ TLR4, ↓ p65 mRNA	[398]
Sodium butyrate	C57Bl/6 mice; HFD (45% fat, 12 weeks); *n* = 7/group	Sodium butyrate 100 mg/kg/day, gavage, 6 weeks; FBA 212.5 mg/kg/day (equimolar)	Preclinical (animal)	Preliminary (direct mitochondrial H_2_O_2_ and GSH/GSSG measurement; NRF2 pathway; no human data)	↓ H_2_O_2_ yield (mitochondria, *p* < 0.05); ↑ Aconitase activity (*p* < 0.05); ↑ NQO1, ↑ GST, ↑ GSH/GSSG ratio (*p* < 0.05); ↓ TNF-α, ↓ IL-1β, ↓ MCP-1, ↓ LPS; ↑ NRF2 pathway activation	↓ Body weight, ↓ Body lipid; ↑ Energy expenditure; ↑ State 3 respiration, ↑ Proton leak, ↑ Citrate synthase, ↑ Mitochondrial protein mass; ↑ p-AMPK, ↑ p-ACC; ↓ HOMA-IR, ↑ p-AKT, ↑ GLUT2; ↑ Mfn1/Mfn2/Opa1, ↓ Drp1/Fis1	[397]
Sodium butyrate	C57BL/6J mice; HFD (45% fat, 8 weeks)	Sodium butyrate 80 mg/mouse, gavage every other day, 10 days (5 doses)	Preclinical (animal)	Preliminary (no oxidative stress biomarkers directly measured; no human data)	↓ Leptin (plasma, *p* < 0.05); ↑ H3K9Ac on ARβ3 promoter; ↑ GPR43, ↑ p-CREB	↓ Body weight; ↓ Epididymal fat mass and adipocyte size; ↑ ARβ3, ↑ PKA, ↑ ATGL, ↑ p-HSL (Ser563); ↑ PGC-1α (mRNA and protein), ↑ COX4, ↑ mtDNA-encoded genes (ND2, ND4, ND4L, COX1)	[399]
SCFAs (acetate, propionate, butyrate)	C57Bl/6J mice (8-week-old males); HFD (45% fat from palm oil)	SCFAs 5% (*w*/*w*) incorporated into HFD for 12 weeks (prevention) or 6 weeks after 12-week HFD (treatment)	Preclinical (animal)	Preliminary (no oxidative stress biomarkers directly measured; no human data)	↑ UCP2 expression (liver and WAT); ↑ Mitochondrial uncoupling (↑ state 4 respiration); ↑ AMP-to-ATP ratio; ↑ p-AMPK and p-ACC; ↓ malonyl-CoA; ↑ CPT-1 activity; PPARγ-dependent mechanism	↓ Fasting insulin, ↑ GIR, ↑ peripheral glucose uptake; ↓ Plasma NEFA, ↓ Hepatic TG, ↓ Hepatic lipogenesis, ↑ Hepatic β-oxidation; ↓ Body weight gain, ↓ WAT mass, ↓ Adipocyte size, ↓ Leptin; ↑ Energy expenditure, ↓ RER	[400]
Sodium butyrate	Children with obesity (*n* = 54, age 5–17 years, BMI >95th percentile); BAPO trial	Sodium butyrate 20 mg/kg/day (max 800 mg/day) + standard care (Mediterranean diet, 60 min aerobic activity daily) for 6 months	Clinical (RCT, quadruple-blind, placebo-controlled)	Moderate (RCT design; direct inflammatory markers IL-6 and miR-221; small sample size)	↓ IL-6; ↓ miR-221 relative expression; ↓ ghrelin	↓ BMI; ↓ WC; ↓ insulin; ↓ HOMA-IR; ↑ gene richness.	[401]

↓ Decrease; ↑ increase; ↔ no change; i.p. intraperitoneal; WC Waist circumference. Strong requires direct oxidative stress biomarker measurement in human RCTs with an adequate sample size and reproducible findings; Moderate applies to human RCTs with indirect markers or well-controlled preclinical studies with direct biomarker measurement; Limited applies to human studies without direct biomarker measurement, non-randomized designs, or preclinical studies without direct oxidative stress biomarkers; Preliminary applies to preclinical studies with direct biomarker measurement but no human data; and Not Established applies to interventions with failed clinical translation or ex vivo studies only.

## Data Availability

No new data were created or analyzed in this study. Data sharing is not applicable to this article.

## Abbreviations

The following abbreviations are used in this manuscript:

TNF-α	Tumor Necrosis Factor-α
IL-6	Interleukin-6
IL-1β	Interleukin-1β
ROS	Reactive Oxygen Species
IR	Insulin Resistance
MAMs	Mitochondria-Associated ER Membranes
TCA	Tricarboxylic Acid
O^2−^	Superoxide Anion
H_2_O_2_	Hydrogen Peroxide
ETC	Electron Transport Chain
FFAs	Free Fatty Acids
HDAC3	Histone Deacetylase 3
HFD	High-Fat Diet
MafK	MAF bZIP transcription factor K
MFN	Mitofusin 1
NOX	NADPH oxidase
NRF1/2	Nuclear Respiratory Factors 1/2
ERRα	Estrogen-Related Receptor α
PGC-1α	Peroxisome Proliferator-Activated Receptor Gamma Coactivator 1-α
MFN1	Membrane Protein Mitofusin 1
MFN2	Membrane Protein Mitofusin 2
OPA1	Optic Atrophy 1
SOD1	Superoxide Dismutase 1
T2DM	Type 2 Diabetes Mellitus
CRP	C-Reactive Protein
VAT	Visceral Adipose Tissue
WAT	White Adipose Tissue
GPx	Glutathione Peroxidase
HIF-1α	Hypoxia-Inducible Factor 1-α
TLR4	Toll-Like Receptor 4
ER	Endoplasmic Reticulum
UPR	Unfolded Protein Response
8-OHdG	8-Hydroxy-2′-Deoxyguanosine
PON-1	Paraoxonase-1
GGT	Gamma-Glutamyl Transferase
miRNAs	MicroRNAs
OxPLs	Oxidized Phospholipids
Trx2	Thioredoxin 2
LPS	Lipopolysaccharide
